# Single‐Cell Virtual Perturbation Screening Identifies STAT3 as a Key Regulator of Dentinogenesis

**DOI:** 10.1111/cpr.70203

**Published:** 2026-04-15

**Authors:** Yanfei Zhu, Hongyuan Xu, Zijian Zhang, Siyuan Sun, Zihan Huang, Xin Gao, Houwen Pan, Xiangru Huang, Yuanqi Liu, Xinyu Wang, Hanbin Jia, Qinggang Dai, Lingyong Jiang

**Affiliations:** ^1^ Center of Craniofacial Orthodontics, Department of Oral and Cranio‐Maxillofacial Surgery, Shanghai Ninth People's Hospital, Shanghai Jiao Tong University School of Medicine, College of Stomatology, Shanghai Jiao Tong University, National Center for Stomatology, National Clinical Research Center for Oral Diseases, Shanghai Key Laboratory of Stomatology, Shanghai Research Institute of Stomatology Shanghai China; ^2^ The 2nd Dental Center, Shanghai Ninth People's Hospital, Shanghai Jiao Tong University School of Medicine, College of Stomatology, Shanghai Jiao Tong University, National Center for Stomatology, National Clinical Research Center for Oral Diseases, Shanghai Key Laboratory of Stomatology, Shanghai Research Institute of Stomatology Shanghai China

**Keywords:** dentine development, dentinogenesis, in silico perturbation, odontoblast differentiation, STAT3, Wnt signalling pathway, WNT2B

## Abstract

Dentine formation constitutes a physiological process precisely regulated by signal transduction modules governing odontoblast differentiation and mineralisation. First, by constructing a single‐cell transcriptional landscape of odontogenic tissue, we defined EFNB2+ mesenchymal cells as a primary progenitor cluster, marking the origin of the odontogenic lineage. Integrating CellRank‐based fate mapping and SCENIC‐based regulon specificity analysis, we identified signal transducer and activator of transcription 3 (STAT3) as a pivotal transcriptional regulator of the odontoblast lineage. Subsequently, in silico perturbations using CellOracle predicted that *STAT3* ablation disrupted the developmental vector field, redirecting the fate of mesenchymal precursors away from the odontoblast lineage. To substantiate these bioinformatic predictions, functional validation using shRNA‐mediated silencing and pharmacological modulation demonstrated that STAT3 was essential for the proliferation and differentiation capacity of dental mesenchymal cells. Furthermore, we generated conditional knockout mice targeting *Stat3* in *Osterix*‐expressing odontoblast progenitors, which consequently exhibited significant dentine dysplasia. Mechanistically, RNA‐seq and chromatin immunoprecipitation (ChIP) assays revealed that STAT3 directly bound to the *WNT2B* promoter, transcriptionally activating the Wnt/β‐catenin signalling pathway in dental mesenchymal cells. Overexpression of *WNT2B* partially rescued the odontogenic defects induced by STAT3 inactivation. This ‘prediction to verification’ study establishes STAT3 as a critical regulator of dentinogenesis and provides potential therapeutic targets for the treatment of dentine developmental disorders and the advancement of dentine regeneration.

## Graphical abstract

STAT3 promotes odontoblast differentiation in dental mesenchymal cells by transcriptionally regulating *WNT2B* via the canonical Wnt/β‐catenin signalling pathway. These findings elucidate a mechanism underlying dentine development.

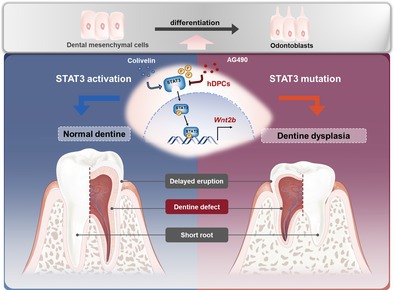

## 1 Introduction

Dentine, the main component of a tooth, is a highly mineralised tissue under enamel, which safeguards the pulp from infection, supports enamel by nutrient transfer and attenuates masticatory forces [1]. During the initial stage of dentine formation, the inner enamel epithelial cells proliferate and migrate in an apical direction. Receiving the signals from epithelial cells, neural crest‐derived mesenchymal cells in the dental papilla and dental pulp differentiate into pre‐odontoblasts [2, 3]. After polarisation, pre‐odontoblasts mature and start to produce extracellular matrix components. This secretion initiates the mineralisation process, ultimately transforming predentin into mature dentine [4, 5]. Throughout the process of dentine formation, the odontoblast differentiation of dental mesenchymal cells plays an indispensable role, which is regulated by a complicated network highly ordered in temporal and spatial sequences [6].

Abnormal molecular signalling during dentine formation leads to dentine dysplasia, characterised by aberrant dentine mineralisation, diminutive roots and rapid tooth attrition [7], and it is a common associated manifestation of various congenital syndromes [8]. Pathways including WNT, BMP/TGF‐β, MAPK, NOTCH and NF‐κB have been reported to form interconnected circuits that regulate odontoblast differentiation during dentine formation [9, 10]. Nevertheless, much remains unknown regarding the comprehensive molecular mechanisms that regulate dentinogenesis. In order to obtain a global view of the transcriptional regulation underlying dentine development, we integrated single‐cell transcriptomic datasets from odontogenic tissue. Subsequent analysis using both CellRank and the regulon specificity score (RSS) identified signal transducer and activator of transcription 3 (STAT3) as a key driver gene for odontogenic lineage.

STAT3, a member of the JAK–STAT signalling family, functions as a nuclear messenger that transduces extracellular signals from cytokines, hormones and growth factors into transcriptional activation programs [11]. STAT3 has been reported to be a pivotal molecular regulator in both immune response and tumorigenic progression. Emerging evidence in recent years has increasingly implicated its involvement in bone homeostasis maintenance [12, 13, 14]. Odontogenesis and osteogenesis share some similarities in originating from mesenchymal precursors and involving coordinated matrix deposition and mineralisation cascades. Despite these shared mechanistic features, the specific regulatory functions of STAT3 in tooth development remain poorly characterised.

Herein, this study implemented a dual‐validation integrating bioinformatics prediction (‘virtual knockout’) with biological verification (‘experimental knockout’), demonstrating the pivotal role of STAT3 in dentine development. STAT3 function was validated using a combination of lentiviral‐mediated knockdown and pharmacological modulation in vitro, which was further corroborated in vivo through conditional knockout mice, where *Stat3* was targeted in *Osterix* (*Osx*)‐expressing odontoblast progenitors via the *Cre‐LoxP* system. Finally, the molecular mechanisms underlying STAT3‐regulated dentinogenesis were elucidated.

## 2 Materials and Methods

### 2.1 Ethical Approval Statement

All animal experimental protocols were reviewed and approved by the Institutional Animal Care and Use Committee of the Shanghai Ninth People's Hospital, Shanghai Jiao Tong University School of Medicine (Ethical Approval No. HKDL [2018]386). All procedures were conducted in compliance with the ARRIVE guidelines.

### 2.2 Single‐Cell Transcriptomic and Pseudotemporal Analysis

Single‐cell data derived from human odontogenic tissues were sourced from the GEO dataset (GSE146123) [15]. All transcriptomic data underwent quality control and normalisation using the Scanpy pipeline [16]. Batch correction across samples was performed with scVI [17]. Unsupervised clustering was applied on a k‐nearest neighbour graph using the Leiden community detection algorithm. Nonlinear dimensionality reduction was conducted with UMAP. Branching lineages and pseudotemporal ordering within the single‐cell data were inferred using Slingshot [18]. Cell differentiation potential was assessed with the CytoTRACE2 framework [19], which utilises transcriptional diversity as an interpretable metric to delineate developmental potency gradients. Directed fate mapping was performed with CellRank to estimate the absorption probability of cells towards potential terminal states [20], identify initial and terminal cell states and visualise the fate landscape through circular projection.

### 2.3 Transcriptional Regulatory Network Inference and Regulon Specificity Scoring

To systematically dissect the upstream transcription factor (TF) regulatory networks driving fate decisions in mesenchymal subpopulations, we employed the SCENIC framework for single‐cell gene regulatory network (GRN) reconstruction and regulon activity analysis [21]. Specifically, the scalable pySCENIC workflow was implemented to construct gene co‐expression modules, refine regulons by integrating motif/enhancer information, and evaluate activity using AUCell. The RSS was calculated for each TF regulon to quantify its specificity pattern across distinct cell subgroups. Key candidate regulators were subsequently prioritised based on convergent evidence from RSS ranking and fate probabilities, aiming to explain the coherence in lineage differentiation directions.

### 2.4 In Silico Gene Knockout and Network Perturbation Analysis

To evaluate the causal role of key TFs in cell state transition and lineage commitment, in silico gene knockout analysis was performed using CellOracle [22]. Built upon the GRN inferred from single‐cell data, CellOracle simulates the impact of perturbing a specific TF on the transcriptional network and the cellular ‘vector field’, thereby predicting alterations in fate trajectory and potential differentiation blockade. In this study, we focused on assessing the effect of a virtual STAT3 knockout on odontoblast lineage commitment, validating its consistency with trajectory and fate probability results. Furthermore, scTenifoldKnk was utilised to execute a virtual knockout of the target gene, construct wild‐type and ‘virtual KO’ single‐cell GRNs, and identify differentially regulated genes to quantify perturbation effects at the network level [23]. Standard over‐representation analysis was performed on the gene sets associated with network perturbation, mapping them to Gene Ontology (GO) terms and KEGG pathways [24].

### 2.5 Mice

All experimental mice were maintained in a controlled specific pathogen‐free (SPF) condition. *Stat3* ^*fl/fl*^ strain and *Osx* ^*Cre*^ strain were purchased from the Jackson Laboratory (No. 016923, No. 006361). All these mice were maintained on the C57BL/6 background. *Stat3* ^*fl/fl*^ mice were crossed with *Osx* ^*Cre*^ mice to generate *Stat3* ^*fl/fl*^ *;Osx* ^*Cre*^ knockout mice. *Stat3* ^*fl/fl*^ littermates were used as controls.

### 2.6 Human Dental Pulp Cells Isolation and Culture

The human dental pulp cells (hDPCs) isolation and culture procedure received approval from the Institutional Review Board of Shanghai Ninth People's Hospital, Shanghai Jiao Tong University School of Medicine (Ethical Approval No. SH9H‐2023‐TK381‐1). Human dental pulp tissues were collected from extracted healthy third molar germs following informed consent, finely minced with ophthalmic scissors, and enzymatically digested in a solution containing 1 mg/mL Collagenase I and 2 mg/mL Dispase II (Sigma‐Aldrich, MO, USA) in α‐MEM (Corning, NY, USA) at 37°C for 30 min. The digested fragments were subsequently cultured in α‐MEM supplemented with 10% foetal bovine serum (Gibco, NY, USA) and 1% Penicillin/Streptomycin (Thermo Fisher Scientific, MA, USA).

### 2.7 Statistical Analysis

Statistical analyses were performed using GraphPad Prism 10.4.1 software (GraphPad Software, San Diego, CA, USA). All experiments were independently repeated in at least three biological replicates. Quantitative data were expressed as the mean ± standard deviation (SD). The Shapiro–Wilk (SW) test was used to verify the normality of data. Student's *t*‐test was applied for statistical comparisons between two groups. One‐way analysis of variance (ANOVA) was used for multi‐group comparisons. *p*‐value < 0.05 was considered statistically significant.

Additional experimental procedures relevant to this study are documented in the Supplementary experimental procedures.

## 3 Results

### 3.1 Single‐Cell Transcriptional Landscape of Human Odontogenic Tissue

**FIGURE 1:**
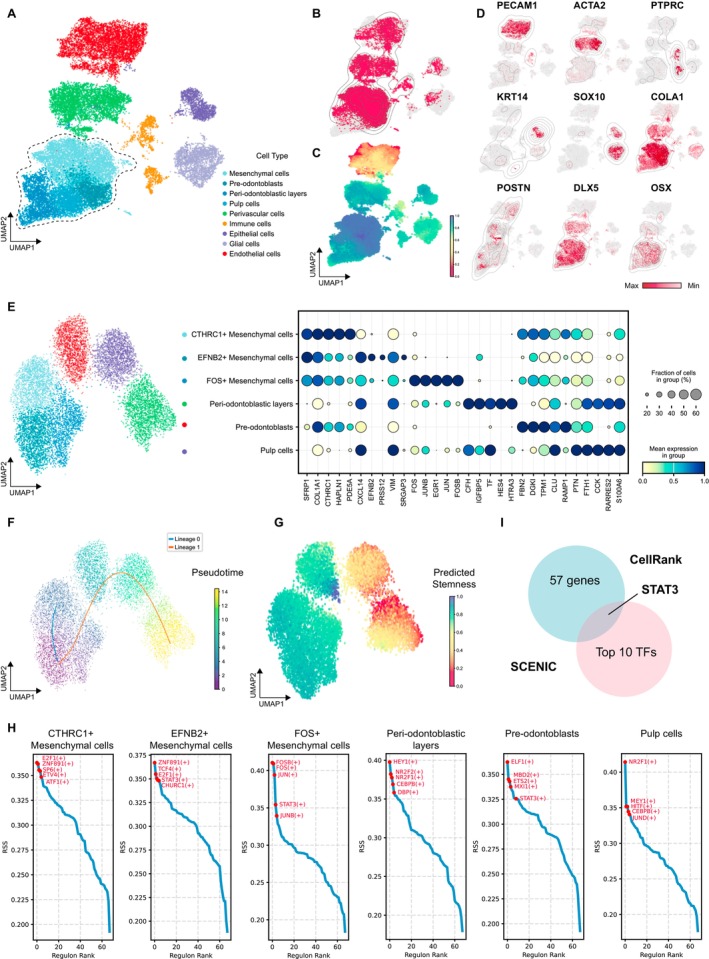
Single‐cell transcriptional landscape of odontogenic tissue. (A) UMAP of 24,177 cells clustered. (B) Distribution of dental papilla tissue aligned with single‐cell annotations. (C) CytoTRACE2 differentiation scores projected onto the UMAP. (D) Marker gene expression supporting cell‐type assignment. (E) Subclustering of odontogenic mesenchymal lineage cells showing six subgroups. (F) Slingshot pseudotime trajectory with a bifurcation from EFNB2+ mesenchymal cells. (G) Stemness scores across subgroups. (H) SCENIC regulon specificity score (RSS) ranking highlighting pre‐odontoblast–specific regulons. (I) STAT3 identified as a key regulator by intersecting CellRank and SCENIC results.

To comprehensively characterise the cellular heterogeneity within the developing dental microenvironment, we performed unsupervised clustering on the quality‐controlled and integrated human‐derived single‐cell transcriptomic dataset, which comprised a total of 24,177 high‐quality cells for downstream analysis. Using the Leiden community detection algorithm (Figure S1A) in combination with Uniform Manifold Approximation and Projection (UMAP) for dimensionality reduction, we partitioned the cells into distinct transcriptional clusters (Figure 1A). The UMAP projection revealed clear community structures among the cells. By integrating information from the spatial distribution of dental papilla tissue (Figures 1B and S1B), the differentiation scores estimated by the CytoTRACE2 algorithm (Figures 1C and S1C), and differential expression analysis based on canonical marker genes (Figures 1D and S1D), we identified nine major cell types within this tissue. These include: endothelial cells (*n* = 5128; 21.2%), mesenchymal cells (*n* = 5000; 20.7%), perivascular cells (*n* = 4079; 16.9%), glial cells (*n* = 2098; 8.7%), peri‐odontoblastic layer cells (*n* = 2076; 8.6%), pulp cells (*n* = 1805; 7.5%), pre‐odontoblasts (*n* = 1711; 7.1%), epithelial cells (*n* = 1228; 5.1%) and immune cells (*n* = 1052; 4.4%). To further investigate the heterogeneity of odontogenic mesenchymal lineage cells, we performed sub‐clustering analysis on the selected 10,353 cells. Based on differential gene expression signatures, these cells were stratified into six distinct subgroups (Figures 1E and S1E). The mesenchymal cells could be further subdivided into three subpopulations: CTHRC1+ mesenchymal cells, EFNB2+ mesenchymal cells and FOS+ mesenchymal cells, suggesting their potential functional divergence in microenvironmental regulation and tissue remodelling.

To reconstruct the differentiation trajectory of the odontogenic mesenchymal lineage, we inferred cellular pseudotime using the Slingshot algorithm. Trajectory analysis revealed that EFNB2+ mesenchymal cells resided at the root of the developmental trajectory, which subsequently bifurcated into two major branches (Figure 1F). One branch was directed towards pre‐odontoblasts and peri‐odontoblastic layers, indicating the odontogenic differentiation path, while the other branch led to distinct mesenchymal cell subpopulations, reflecting heterogeneous differentiation towards stromal remodelling. To validate this differentiation potential, we evaluated the stemness score of each subgroup using the CytoTRACE2 algorithm (Figure S1F). The results (Figure 1G) showed that EFNB2+ mesenchymal cells possessed the highest stemness score. This alignment with the Slingshot‐inferred trajectory further confirmed the status of EFNB2+ mesenchymal cells as the primary progenitor cells within this microenvironment.

To systematically dissect the transcriptional regulatory networks driving the fate determination of mesenchymal subgroups, we integrated CellRank dynamics analysis with SCENIC‐based TF inference. CellRank circular projection (Figure S1G) identified EFNB2+ mesenchymal cells as the multipotent starting state, capable of differentiating into multiple terminal states, with fate probabilities highly correlated with lineage‐specific marker expression. Subsequently, key driver genes for the odontogenic lineage were identified using CellRank (Table S1). In parallel, we calculated the RSS for each TF using the SCENIC algorithm. The RSS rank plot (Figure 1H, Table S2) demonstrated that multiple TF regulons exhibited exceptionally high specific activity within the pre‐odontoblast subgroup. Considering the convergent evidence from both fate probability and regulon specificity, we took the intersection of results from the two methods and identified STAT3 as a key regulator (Figure 1I). Concurrently, the expression of STAT3 exhibited dynamic changes along the pseudotime (Figure S1H), with a significant increase observed during the early differentiation of pre‐odontoblasts. This finding strongly supports STAT3 as a critical regulator of odontoblast differentiation, potentially promoting the transition of precursor cells into mature odontoblasts.

### 3.2 In Silico *STAT3* Knockout Perturbs Fate of Odontoblast Lineage

**FIGURE 2:**
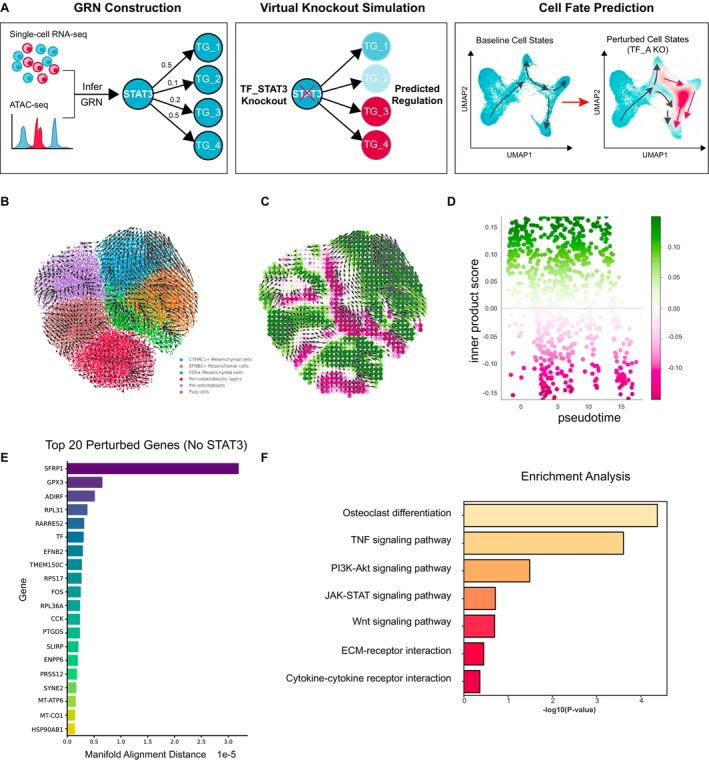
In silico *STAT3* knockout perturbs the fate of odontoblast lineage. (A) Workflow of CellOracle‐based virtual *STAT3* knockout. (B–D) Vector field and trajectory shifts indicating reduced commitment towards the pre‐odontoblast lineage. (E) ScTenifoldKnk‐validated genes with significant network perturbation after *STAT3* knockout. (F) Functional enrichment of perturbed genes.

To investigate the functional impact of STAT3, we performed in silico gene knockout analysis using CellOracle (Figure 2A). The virtual knockout of *STAT3* induced significant kinetic remodelling within the transcriptional regulatory network. The perturbed vector field revealed an overall shift in the predicted migration direction of cell states away from pre‐odontoblasts (Figure 2B–D), suggesting that STAT3 is critically required for driving the fate commitment of mesenchymal precursors towards the odontoblast lineage. This finding aligns with our previous conclusions and provides functional validation for the central role of STAT3 in the odontoblast differentiation. To further validate and quantify the network perturbation effect, we employed scTenifoldKnk. The results confirmed that perturbation of STAT3 was accompanied by significant changes in the expression of multiple development‐related genes (Figure 2E). Subsequent functional enrichment analysis showed that the affected genes were significantly enriched in pathways closely associated with cell differentiation and odontogenic metabolism, such as the Wnt signalling pathway (Figure 2F).

### 3.3 Ablation of *STAT3* in Dental Mesenchymal Cells Leads to Impaired Odontoblast Differentiation

**FIGURE 3:**
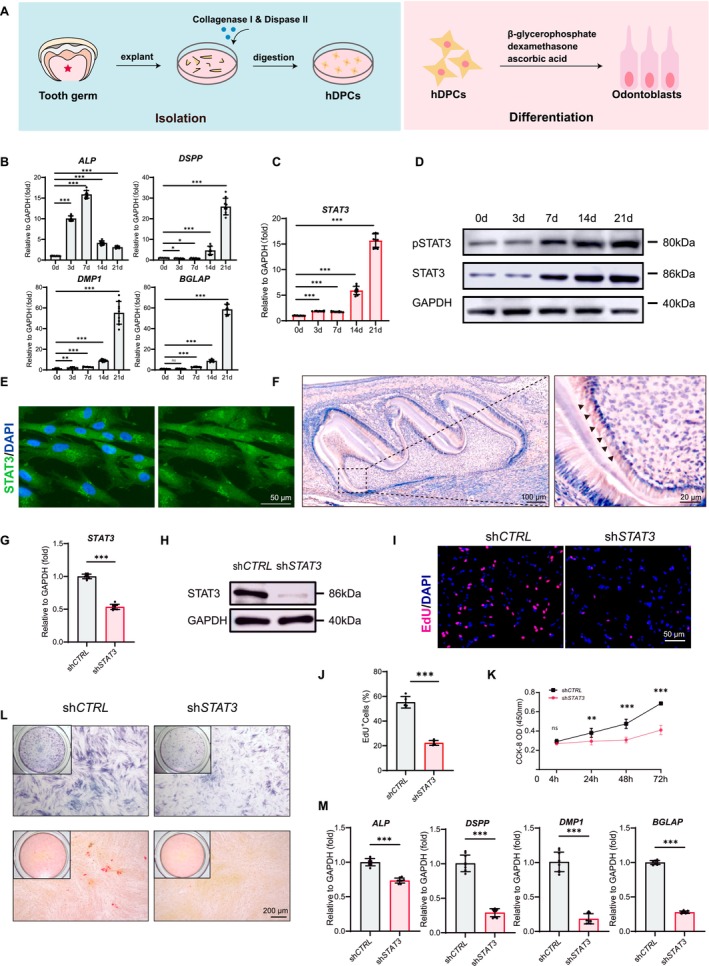
Expression of STAT3 in odontogenic tissue is required for odontoblast differentiation. (A) Schematic diagram illustrating the isolation of human dental pulp cells (hDPCs) and odontogenic induction process. (B) The relative messenger RNA (mRNA) expression levels of odontoblast‐related genes (*ALP, DSPP*, *DMP1* and *BGLAP*) in hDPCs cultured in odontogenic medium for 0, 3, 7, 14 and 21 days. (C) The mRNA expression levels of *STAT3* in hDPCs cultured in odontogenic medium for 0, 3, 7, 14 and 21 days. (D) Western blotting of STAT3 protein expression in hDPCs during odontogenic induction at 0, 3, 7, 14 and 21 days. (E) Immunofluorescence staining of STAT3 in induced odontoblasts. (F) Immunohistochemical staining of STAT3 in mandibular first molar from 1‐week‐old mice. Arrows indicate STAT3‐positive odontoblasts. (G) The mRNA levels of *STAT3* in hDPCs infected with *STAT3*‐deletion lentivirus and control EGFP‐expressing lentivirus. (H) Western blotting of STAT3 in hDPCs infected with *STAT3*‐deletion lentivirus and control lentivirus. (I) Cell Counting Kit‐8 (CCK‐8) assay to analyse the proliferation of *STAT3*‐silenced hDPCs and control cells. (J) EdU immunofluorescence staining of *STAT3*‐silenced hDPCs and control cells. (K) Quantitative analysis of EdU immunofluorescence staining, *n* = 5. (L) ALP and ARS staining images for *STAT3*‐silenced hDPCs and control cells after odontogenic induction. (M) The mRNA levels of odontoblast‐related genes (*ALP, DSPP*, *DMP1* and *BGLAP*) in *STAT3*‐silenced hDPCs and control cells after odontogenic induction. Error bars represent mean ± SD. *p < 0.05; **p < 0.01; ***p < 0.001; ns, not significant (*p* > 0.05).

To substantiate the regulatory role of STAT3 predicted by our in silico prediction, we first characterised its expression pattern during dentinogenesis. The hDPCs were isolated and cultured in odontogenic induction medium to induce differentiation into odontoblasts (Figure 3A). Quantitative analysis showed significant upregulation of odontogenic markers, including *alkaline phosphatase* (*ALP*), *dentine sialophosphoprotein* (*DSPP*), *dentine matrix protein 1* (*DMP1*) and *bone gamma‐carboxyglutamate protein* (*BGLAP*) during odontoblast differentiation (Figure 3B). Notably, both STAT3 expression levels and phosphorylation status were progressively elevated during this process (Figure 3C,D). Immunofluorescence analysis demonstrated the subcellular localisation of STAT3 in hDPCs (Figure 3E). Immunohistochemical staining of mandibular first molars from 1‐week‐old mice confirmed robust STAT3 expression in odontoblast lineage cells (Figure 3F). These findings indicate that STAT3 is ubiquitously expressed in odontogenic tissues and dynamically activated during odontoblast differentiation, implicating its functional involvement in dentinogenesis.

To further delineate the necessity of STAT3 in odontoblast lineage, we established a loss‐of‐function model in hDPCs using shRNA‐mediated gene silencing. Knockdown efficiency was confirmed at both mRNA and protein levels (Figure 3G,H). Functional assessments revealed that *STAT3* depletion significantly compromised hDPCs proliferation, as shown by Cell Counting Kit‐8 (CCK‐8) assay and EdU^+^ cell immunofluorescence staining (Figure 3I–K). Moreover, *STAT3*‐deficient hDPCs exhibited multifunctional defects in odontoblast differentiation, including reduced ALP activity and diminished mineralisation capacity (Figure 3L). These changes were consistent with downregulated expression of odontoblast markers, including *ALP*, *DSPP*, *DMP1* and *BGLAP* in *STAT3* knockdown hDPCs at the transcriptional level (Figure 3M). Collectively, these in vitro results indicate that *STAT3* ablation compromises the odontogenic potential of hDPCs, thereby disrupting the orchestrated process of dentinogenesis.

### 3.4 Conditional Deletion of *Stat3* in Odontoblast Progenitors Results in Dentine Dysplasia

**FIGURE 4:**
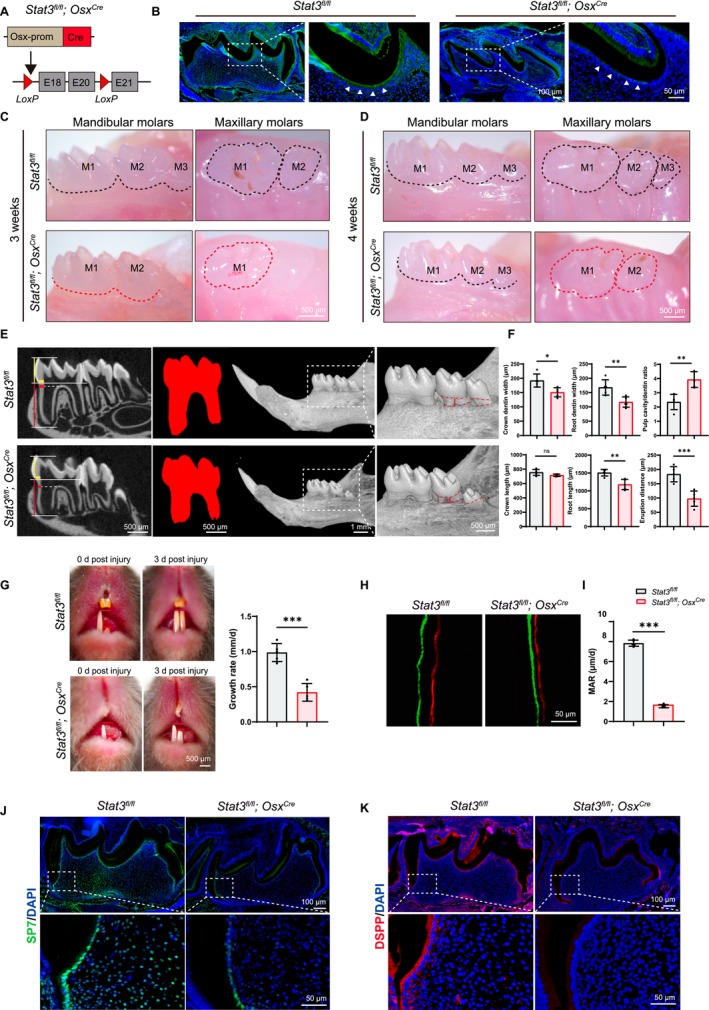
Conditional deletion of *Stat3* in odontoblast progenitors results in dentine dysplasia. (A) Schematic diagram of *Stat3* deletion in Osterix (*Osx*)‐expressing odontoblast progenitors. (B) Anti‐STAT3 immunofluorescence staining of mandibular first molars from 1‐week‐old *Stat3* ^*fl/fl*^ *;Osx* ^*Cre*^ and *Stat3* ^*fl/fl*^ mice. Arrows indicate the odontoblast layer. (C) Stereomicroscopic images of mandibular and maxillary molars from 3‐week‐old *Stat3* ^*fl/fl*^ *;Osx* ^*Cre*^ and *Stat3* ^*fl/fl*^ mice. (D) Stereomicroscopic images of mandibular and maxillary molars from 4‐week‐old *Stat3* ^*fl/fl*^ *;Osx* ^*Cre*^ and *Stat3* ^*fl/fl*^ mice. (E) Micro‐CT images of mandibular molars from 4‐week‐old *Stat3* ^*fl/fl*^ *;Osx* ^*Cre*^ and *Stat3* ^*fl/fl*^ mice. (F) Quantitative analysis of micro‐CT images, including the mandibular first molar crown dentine width, root dentine width, root length, crown length, pulp cavity/dentine ratio, and eruption distance of the mandibular second molar, *n* = 5. (G) Representative images and quantitative analysis of incisors from 4‐week‐old *Stat3* ^*fl/fl*^ *;Osx* ^*Cre*^ and *Stat3* ^*fl/fl*^ mice immediately after incisor injury and 3 days post‐injury, *n* = 5. (H) Double labelling images of mandibular first molars from 3‐week‐old *Stat3* ^*fl/fl*^ *;Osx* ^*Cre*^ and *Stat3* ^*fl/fl*^ mice. (I) Quantitative analysis of dentine mineral apposition rate (MAR), *n* = 5. (J) Anti‐SP7 immunofluorescence staining of mandibular first molars from 1‐week‐old *Stat3* ^*fl/fl*^ *;Osx* ^*Cre*^ and *Stat3* ^*fl/fl*^ mice. (K) Anti‐DSPP immunofluorescence staining of mandibular first molars from 1‐week‐old *Stat3* ^*fl/fl*^ *;Osx* ^*Cre*^ and *Stat3* ^*fl/fl*^ mice. Error bars represent mean ± SD. *p < 0.05; **p < 0.01; ***p < 0.001; ns, not significant (*p* > 0.05).

To validate the in vivo relevance of STAT3 inactivation in odontoblast lineage to dentine development defects, we generated odontoblast progenitor‐specific *Stat3* conditional knockout mice (*Stat3* ^*fl/fl*^ *;Osx* ^*Cre*^) by crossing *Stat3* ^*fl/fl*^ mice with *Osx* ^*Cre*^ mice, in which *Cre* recombinase expression is specifically restricted to early odontoblast precursors (Figure 4A) [25, 26]. Immunofluorescence staining confirmed efficient *Stat3* depletion in odontoblast lineage (Figure 4B). Stereomicroscopic analysis revealed that *Stat3* ^*fl/fl*^ *;Osx* ^*Cre*^ mice developed significant tooth eruption retardation in maxillary second molars at postnatal Week 3 (Figure 4C) and in maxillary third molars at postnatal Week 4 compared to control littermates (*Stat3* ^*fl/fl*^ mice) (Figure 4D). Micro‐CT reconstructions of 4‐week‐old mandibular molars showed multilevel dental defects in *Stat3* ^*fl/fl*^ *;Osx* ^*Cre*^ mice, including reduced dentine width, shortened roots and delayed eruption of third molars (Figure 4E). Quantitative analysis confirmed significant reductions in multiple parameters of *Stat3* ^*fl/fl*^ *;Osx* ^*Cre*^ mice at 4 weeks, including crown dentine width, root dentine width, as well as root length of mandibular first molars and eruption distance of mandibular second molars, while crown length showed no intergroup difference. The pulp cavity/dentine ratio was significantly increased in mutants (Figure 4F). In contrast, enamel thickness did not differ between mutant and control mice, indicating that the observed dental defects were restricted to the dentine (Figure S2). Longitudinal micro‐CT assessment (Weeks 3, 4 and 8) across genotypes (*Stat3* ^*fl/fl*^ *;Osx* ^*Cre*^, *Stat3* ^*fl/+*^ *;Osx* ^*Cre*^ and *Stat3* ^*fl/fl*^) revealed gene dosage‐dependent developmental dentine defects (Figure S3). *Stat3* homozygous mutants exhibited persistently weakened dentine and short roots throughout odontogenesis and after maturation. Heterozygote mice displayed attenuated phenotypes, confirming gene dosage effects. Beyond impaired dentine formation, incisor injury assays demonstrated that deletion of *Stat3* in odontoblast progenitors significantly compromised tooth reparative capacity. The quantitative analysis confirmed markedly reduced growth rates in injured incisors of *Stat3* ^*fl/fl*^ *;Osx* ^*Cre*^ mice (Figure 4G).

To elucidate the mechanism underlying *Stat3* ablation induced dentine defects, we first performed histological analysis of mandibular first molars using H&E staining. The results confirmed the dentine structural defects, including significantly reduced pre‐dentine secretion and dentine formation in 1‐ and 3‐week‐old *Stat3* ^*fl/fl*^ *;Osx* ^*Cre*^ mice versus controls (Figure S4A,B). Dynamic mineralisation assessment via calcein‐alizarin red S (ARS) double labelling showed a statistically significant reduction in mineral apposition rate within the dentine layer of *Stat3* mutants (Figure 4H,I). Furthermore, immunofluorescence staining of odontogenic markers SP7 and DSPP showed attenuated expression patterns in 1‐week‐old *Stat3* ^*fl/fl*^ *;Osx* ^*Cre*^ mice relative to controls (Figure 4J,K). Collectively, these results demonstrated that the reduced dentine thickness and shortened root phenotypes in *Stat3* conditional knockout mice originated from impaired dentinogenesis.

### 3.5 STAT3 Acts as a Potential Pharmacological Target in Dentinogenesis

**FIGURE 5:**
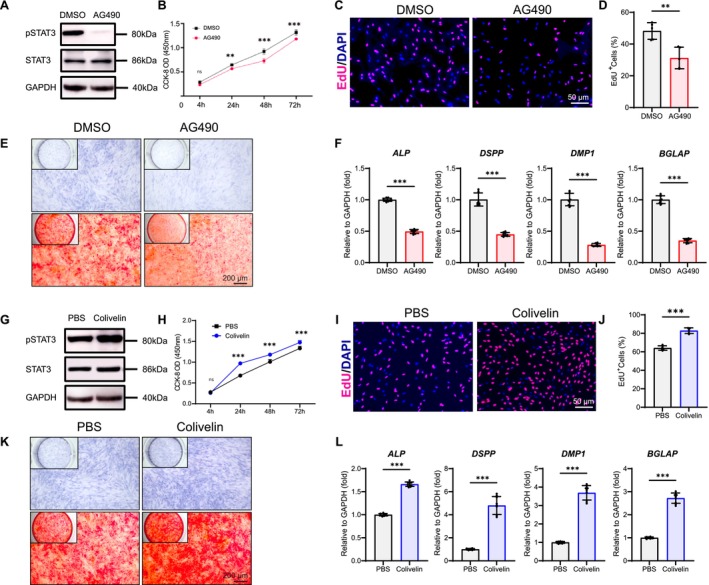
STAT3 acts as a potential pharmacological target in dentinogenesis. (A) Western blotting of STAT3 and pSTAT3 in hDPCs treated with AG490 and DMSO. (B) CCK‐8 assay to analyse the proliferation of hDPCs treated with AG490 and DMSO. (C) EdU immunofluorescence staining of hDPCs treated with AG490 and DMSO. (D) Quantitative analysis of EdU immunofluorescence staining, *n* = 5. (E) ALP and ARS staining images of hDPCs treated with AG490 and DMSO after odontogenic induction. (F) The mRNA levels of odontoblast‐related genes (*ALP, DSPP*, *DMP1* and *BGLAP*) in hDPCs treated with AG490 and DMSO after odontogenic induction. (G) Western blotting of STAT3 and pSTAT3 in hDPCs treated with colivelin and PBS. (H) CCK‐8 assay to analyse the proliferation of hDPCs treated with colivelin and PBS. (I) EdU immunofluorescence staining of hDPCs treated with colivelin and PBS. (J) Quantitative analysis of EdU immunofluorescence staining, *n* = 5. (K) ALP and ARS staining images of hDPCs treated with colivelin and PBS after odontogenic induction. (L) The mRNA levels of odontoblast‐related genes (*ALP, DSPP*, *DMP1* and *BGLAP*) in hDPCs treated with colivelin and PBS after odontogenic induction. Error bars represent mean ± SD. **p < 0.01; ***p < 0.001; ns, not significant (*p* > 0.05).

Functioning as both a TF and signalling target, STAT3 orchestrates downstream gene expression and also serves as a substrate of multiple signalling pathways and pharmacological agents through phosphorylation. To comprehensively assess the mechanistic regulation of STAT3 in hDPCs beyond mere gene expression modulation, we pharmacologically modulated its activation status. AG490, a JAK2 inhibitor, has been reported to block STAT3 phosphorylation. The phosphorylation level of STAT3 in hDPCs was confirmed to be inhibited by exogenous AG490 at a concentration of 50 μM (Figure 5A). CCK‐8 assay and EdU^+^ cell immunofluorescence staining demonstrated that inhibiting STAT3 activation significantly attenuated proliferation of hDPCs (Figure 5B–D). Additionally, AG490‐treated hDPCs exhibited impaired odontoblast differentiation, as evidenced by reduced ALP activity, diminished mineralisation capacity (Figure 5E), and downregulation of odontoblast‐related markers expression (Figure 5F).

Colivelin, a neuroprotective peptide, has been reported to induce STAT3 phosphorylation. Treating hDPCs with exogenous colivelin at a concentration of 1 nM could significantly enhance STAT3 phosphorylation (Figure 5G). Consistent with this finding, pharmacological activation of STAT3 promoted both proliferation and odontoblast differentiation capabilities of hDPCs (Figure 5H–L).

Pharmacological inhibition of STAT3 phosphorylation impaired proliferation and odontoblast differentiation of hDPCs, whereas its activation enhanced these cellular processes. These findings suggested that STAT3 regulated odontoblast differentiation in hDPCs and might serve as a therapeutic target for modulating dentinogenesis.

### 3.6 STAT3 Modulates Odontoblast Differentiation Through Transcriptional Regulation of *WNT2B*

**FIGURE 6:**
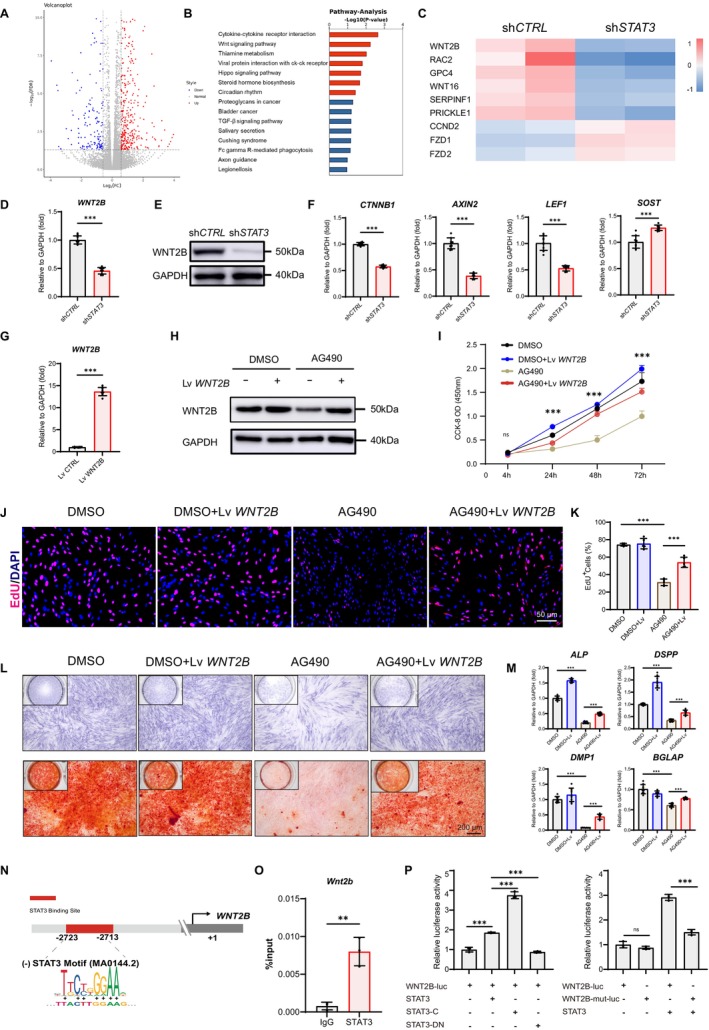
STAT3 modulates odontoblast differentiation through transcriptional regulation of *WNT2B*. (A) Volcano plot displaying globally differentially expressed genes in the *STAT3*‐deleted hDPCs group and control group. A two‐sided test was performed. Blue points represent downregulated genes and red points represent upregulated genes. (B) Pathway enrichment analysis is visualised in a chord plot. (C) Heatmap analysis of Wnt signalling pathway‐related genes. (D) The mRNA levels of *WNT2B* in hDPCs infected with *STAT3*‐deletion lentivirus and control lentivirus. (E) Western blotting of WNT2B in hDPCs infected with *STAT3*‐deletion lentivirus and control lentivirus. (F) The mRNA levels of canonical Wnt signalling pathway related‐genes, including *CTNNB1*, *AXIN2* and *LEF1*, as well as the Wnt inhibitor *SOST*, in hDPCs infected with *STAT3*‐deletion lentivirus and control lentivirus. (G) The mRNA levels of *WNT2B* in hDPCs infected with *WNT2B*‐overexpressing lentivirus and control lentivirus. (H) Western blotting of WNT2B in STAT3‐inactivated hDPCs infected with *WNT2B*‐overexpressing lentivirus and control lentivirus. (I) CCK‐8 assay to analyse the proliferation of STAT3‐inactivated hDPCs infected with *WNT2B*‐overexpressing lentivirus and control lentivirus. (J) EdU immunofluorescence staining of STAT3‐inactivated hDPCs infected with *WNT2B*‐overexpressing lentivirus and control lentivirus. (K) Quantitative analysis of EdU immunofluorescence staining, *n* = 5. (L) ALP and ARS staining images of STAT3‐inactivated hDPCs infected with *WNT2B*‐overexpressing lentivirus and control lentivirus after odontogenic induction. (M) The mRNA levels of odontoblast‐related genes (*ALP, DSPP*, *DMP1* and *BGLAP*) in STAT3‐inactivated hDPCs infected with *WNT2B*‐overexpressing lentivirus and control lentivirus after odontogenic induction. (N) Predicted STAT3 binding site on the *WNT2B* promoter region. (O) Chromatin immunoprecipitation (ChIP) assay showing the binding of STAT3 to the *Wnt2b* promoter in the C3H10T1/2 cell line, *n* = 3. (P) Luciferase assays to evaluate the effects of STAT3, dominant‐negative mutation of STAT3 (STAT3‐DN), and constitutively‐active STAT3 (STAT3‐C), as well as the effect of mutating the STAT3 binding site on the activity of the *WNT2B* promoter in the HEK293T cell line, *n* = 3. Error bars represent mean ± SD. **p < 0.01; ***p < 0.001; ns, not significant (*p* > 0.05).

To map STAT3‐regulated transcriptional networks in hDPCs, we first established *STAT3*‐knockdown (*shSTAT3*) and control (*shCTRL*) cell lines via shRNA‐mediated silencing. These cells were then subjected to RNA‐seq analysis for comparison. Transcriptome profiling identified 617 differentially expressed genes (DEGs) meeting stringent criteria (*p* < 0.05, |log_2_FC| > 0.585), with 426 genes upregulated and 191 genes downregulated upon *STAT3* knockdown (Figure 6A). GO and pathway analyses on the set of significantly downregulated genes identified pronounced enrichment in biological processes, including cytokine‐cytokine receptor interaction, Wnt signalling pathway, and thiamine metabolism (Figure 6B). Our earlier finding from the in silico *STAT3* knockout analysis and subsequent functional enrichment analysis also identified significant enrichment of affected genes in the Wnt signalling pathway (Figure 2F), which was consistent with this observation. Moreover, given the established role of Wnt signalling in odontogenesis [27, 28], we specifically analysed Wnt‐related DEGs. Hierarchical clustering revealed synchronous downregulation of critical Wnt pathway components, including ligands (WNT2B, WNT16), receptors (GPC4), intracellular transducers (RAC2, PRICKLE1) and an extracellular matrix regulator (SERPINF1) (Figure 6C). Among these, WNT2B has been reported to be functionally associated with mineralisation and mesenchymal stem cell differentiation [29]. Validation at both mRNA and protein levels (Figure 6D,E) indicated that *WNT2B* was the most likely target gene of STAT3 in hDPCs. The suppressed expression of canonical Wnt pathway‐related genes, such as *CTNNB1*, *AXIN2* and *LEF1*, coupled with upregulation of the Wnt inhibitor *SOST* in *STAT3*‐knockdown hDPCs further indicated that WNT2B regulated odontoblast differentiation through the canonical Wnt signalling pathway (Figure 6F).

To confirm WNT2B as a STAT3 downstream effector mediating odontoblast differentiation, we constructed lentiviral vectors to overexpress *WNT2B* (Lv‐*WNT2B*) in hDPCs and used AG490 to generate STAT3‐inactivated hDPCs for subsequent functional assays. Lv‐*WNT2B* transduction effectively upregulated the expression of *WNT2B* in hDPCs (Figure 6G) and restored the protein levels of WNT2B in hDPCs treated with AG490 (Figure 6H). Furthermore, *WNT2B* overexpression partially rescued the proliferation deficit in STAT3‐inactivated hDPCs (Figure 6I–K). The impaired odontoblast differentiation capacity was also partially restored by *WNT2B* overexpression in STAT3‐inactivated hDPCs, as evidenced by recovered ALP activity and mineralisation potential (Figure 6L), and upregulated expression of odontoblast‐related markers compared with control groups (Figure 6M).

To investigate the regulatory mechanisms of STAT3 on *WNT2B* expression during odontoblast differentiation, we used JASPAR (CORE vertebrate database) for prediction of TF binding sites and identified a STAT3 binding motif within the *WNT2B* promoter region (Figure 6N). Chromatin immunoprecipitation (ChIP) assay indicated that STAT3 directly bound to the *WNT2B* promoter (Figure 6O). Then, we co‐transfected the STAT3 expression vector, constitutively active STAT3 (STAT3‐C), and dominant‐negative STAT3 mutant (STAT3‐DN) separately with *WNT2B* promoter‐driven luciferase reporters. As shown in Figure 6P, both wild‐type STAT3 and STAT3‐C enhanced *WNT2B* promoter activity, whereas STAT3‐DN showed no transactivation capacity. Furthermore, we generated a *WNT2B* promoter mutant (WNT2B‐mut) by deleting the predicted STAT3 binding motif and verified the specificity of STAT3 binding to the *WNT2B* promoter. Together, these findings indicated that STAT3 regulated odontoblast differentiation by directly binding to and transcriptionally activating *WNT2B* through its conserved DNA‐binding domain.

## 4 Discussion

Dentinogenesis is a sophisticated, cell‐mediated process initiated by the commitment of dental mesenchymal cells to the odontoblast lineage. As terminally differentiated cells, odontoblasts are responsible for the secretion and mineralisation of the organic matrix, a process fundamental to the dentine development and functional capacity of the tooth [30]. Clinically, dentine‐related pathologies, which encompass both congenital developmental disorders (e.g., dentinogenesis imperfecta and dentine dysplasia) and acquired defects due to caries or trauma, represent one of the most prevalent diseases in dentistry [31, 32, 33]. While previous mechanistic research has identified key pathogenic genes such as *DSPP*, *COL1A1*, *COL1A2* and *DMP1* in the pathology of clinical diseases [34, 35, 36, 37], focusing solely on these genetic mutations offers a limited perspective on the broader regulatory landscape. Recent advances in single‐cell transcriptomics, bioinformatic analysis, and gene editing technology have paved the way for a more comprehensive understanding of the regulatory mechanisms in dentinogenesis. By leveraging these technologies, this study implemented an integrated strategy including single‐cell fate mapping, in silico gene perturbations, and experimental validation in both cellular and conditional knockout mouse models to comprehensively elucidate the pivotal role of STAT3 in dentinogenesis, reconciling the gap between bioinformatic prediction and functional verification. This synergy between data‐driven ‘pre‐screening’ and targeted experimental validation provided a high‐efficiency approach for gene discovery and functional characterisation in complex developmental processes.

STAT3, an important JAK/STAT family member, is phosphorylated and activated by IL‐6, EGF and IFN to regulate essential cellular processes including proliferation, differentiation, migration and apoptotic regulation [38, 39]. While accumulating studies have reported that the major biological function of STAT3 involves immune responses and malignancy progression, our work highlights its previously unrecognised function in tooth development. In this study, by synergising CellRank‐based fate probability with SCENIC‐based regulon specificity analysis, we identified STAT3 as a key driver gene governing the transition from multipotent odontoblast progenitors to odontoblasts. The dynamic upregulation of STAT3 along the pseudotemporal axis coincided with the initiation of odontoblastic commitment, a pattern characteristic of factors that trigger lineage‐specific gene expression programs. The CellOracle‐based virtual knockout of *STAT3* resulted in a significant redirection of the developmental vector field, where mesenchymal precursors failed to gravitate towards the odontoblast fate. This kinetic remodelling, further corroborated by scTenifoldKnk analysis, suggests that STAT3 is functionally indispensable for the odontogenic GRN. While the use of CellOracle for in silico perturbation represents an effective approach for predicting concentration changes in cell identity, it has several inherent limitations [22]. First, CellOracle's GRN is inferred from static transcriptomic data and may not fully recapitulate the biological reality. Second, the model assumes linear perturbation propagation through the network, which may underestimate the non‐linear feedback loops and compensatory mechanisms that exist in biological systems. Third, the in silico perturbation represents complete gene ablation and may not accurately reflect physiological conditions characterised by partial loss‐of‐function. Thus, CellOracle should be considered as a powerful screening tool rather than a definitive predictor of cellular behaviour. Furthermore, we generated the *STAT3*‐deficient hDPCs and *Stat3* conditional knockout mice and revealed that deletion of STAT3 in odontoblast progenitor cells impaired odontoblast differentiation leading to compromised dentine formation. Mechanistically, integrated transcriptomic and functional analyses revealed that STAT3 governed odontoblast differentiation via directly activating *WNT2B* transcription, which in turn stimulated canonical Wnt/β‐catenin signalling during dentine development.

Conditional knockout mice provide tissue‐specific insights into STAT3 function within the native microenvironment of developing teeth. Since lineage tracing studies have identified that osterix‐expressing mesenchymal progenitor cells and their descendants are concentrated in dental papilla and pulp, contributing to dentine formation [40, 41], we employed the *Stat3* ^*fl/fl*^ *;Osx* ^*Cre*^ mouse model in this study to conditionally delete *Stat3* in odontoblast precursor cells. The dental phenotypes of the mutant mice were characterised by attenuated dentine thickness (particularly in the root), shortened roots and delayed molar eruption. These observed defects in dentine thickness and root development recapitulate the clinical manifestations of dentine dysplasia type I (DD‐I) [42]. Since root dentine formation facilitates root elongation and tooth eruption, dentine dysplasia disrupts root development, which compromises the eruptive driving force of teeth and leads to delayed eruption [43]. Collectively, these findings suggest that STAT3 is a candidate gene involved in the pathogenesis of dentine dysplasia and a potential therapeutic target for dentinogenesis disorders.

Regarding the downstream regulatory mechanisms of STAT3, recent studies reported that *Stat3* mutations in osteoblasts suppressed Wnt/β‐catenin signalling, leading to bone defects [12, 14]. This pathway is equally critical for odontogenesis [27, 28], as Wnt pathway activation drives β‐catenin/TCF/LEF1‐mediated transcription of odontogenic effectors such as *DSPP* or *DMP1* [44, 45]. Odontoblast‐specific ablation of *Wntless* attenuated Wnt/β‐catenin signalling activity, consequently compromising odontogenic differentiation capacity [46]. These studies are consistent with our in silico perturbation analysis and RNA‐seq findings which revealed that *STAT3* deficiency in DPCs affected the expression of Wnt pathway components. This suggests that STAT3 mediates odontogenesis via Wnt/β‐catenin signalling.

In this study, bioinformatic analysis identified *WNT2B* as a mineralisation and cell differentiation associated gene, which was a downstream regulatory molecule of STAT3 in hDPCs. WNT2B, also known as WNT13, is broadly expressed in the ovary and skin, regulating embryonic development, cell proliferation, differentiation and tissue homeostasis, primarily through the canonical Wnt signalling pathway [47]. Tanaka et al. revealed that *Wnt2b* served as a molecular target of FOXF2 in MSCs mediating bone formation [29]. Purwaningrum et al. reported that IL‐6 enhanced osteogenic differentiation of human periodontal ligament stem cells in a dose‐dependent manner via *WNT2B* [48]. Despite the shared mesenchymal origins of bone and dentine, no relevant research has reported the effect of WNT2B on tooth development. Our study revealed that WNT2B might act as a downstream mediator of STAT3 in regulating dentine formation. RNA‐seq analysis showed that *WNT2B* expression was markedly reduced in *STAT3*‐deficient hDPCs, which was further validated at both mRNA and protein levels. ChIP and luciferase assays confirmed that STAT3 directly bound to and transactivated the *WNT2B* promoter. Furthermore, overexpression of *WNT2B* could partially rescue the proliferation and differentiation defects in STAT3‐inactivated hDPCs. To further validate the functional role of WNT2B in dentine development, future work should employ tissue‐specific *Wnt2b* conditional overexpression mouse models to assess its rescue potential for *Stat3* deficiency‐induced dentinogenesis disorders.

## 5 Conclusion

In conclusion, our study establishes STAT3 as a key transcriptional regulator essential for odontoblast differentiation and dentine formation. Through integrative single‐cell transcriptomic analysis, we identified EFNB2+ mesenchymal cells as the principal progenitors in the dental microenvironment, whose transition into odontoblasts was dependent on the transcriptional activity of STAT3. The deficiency of STAT3, whether induced in silico, in vitro, or via conditional knockout in vivo, impaired odontoblast differentiation and dentine development. Mechanistically, STAT3 activation was essential for the regulation of *WNT2B* in hDPCs, thereby governing odontoblast differentiation via the canonical Wnt/β‐catenin signalling pathway. This research introduces an efficient ‘prediction‐to‐verification’ approach, while also providing new insights into the molecular mechanisms driving odontoblast differentiation and theoretical foundations for dentine regeneration therapies.

## Author Contributions

**Yanfei Zhu:** conceptualisation, investigation, methodology, project administration, writing – original draft, writing – review and editing. **Hongyuan Xu:** data curation, formal analysis, validation. **Zijian Zhang:** data curation, formal analysis, validation. **Siyuan Sun:** data curation, formal analysis, writing – review and editing. **Zihan Huang:** data curation, software. **Xin Gao:** data curation, software. **Houwen Pan:** data curation, software. **Xiangru Huang:** formal analysis. **Yuanqi Liu:** formal analysis. **Xinyu Wang:** formal analysis. **Hanbin Jia:** software, visualisation. All authors have approved the final version for submission. **Qinggang Dai:** conceptualisation, methodology, supervision, project administration. **Lingyong Jiang:** conceptualisation, funding acquisition, resources.

## Conflicts of Interest

The authors declare no conflicts of interest.

## Supporting information


**Data S1:** Supplementary experimental procedures.
**Figure S1:** Supplementary analyses. (A) Leiden clusters. (B) Supplementary spatial distribution overview. (C) CytoTRACE2 scores across clusters. (D) Additional marker gene expression patterns. (E) Marker gene expression patterns of mesenchymal subclusters. (F) CytoTRACE2 scores across clusters of mesenchymal subclusters. (G) CellRank circular projection. (H) Dynamic STAT3 expression along pseudotime.
**Figure S2:** (A) Micro‐CT images with colour mapping based on enamel density in mandibular molars from 4‐week‐old *Stat3* ^*fl/fl*^ *;Osx* ^*Cre*^ and *Stat3* ^*fl/fl*^ mice. (B) Quantitative analysis of enamel thickness in mandibular first molars from 4‐week‐old mice, *n* = 5. Error bars represent mean ± SD. Ns, not significant (*p* > 0.05).
**Figure S3:** Micro‐CT images of mandibular molars from 3‐, 4‐ and 8‐week‐old *Stat3* ^*fl/fl*^ *;Osx* ^*Cre*^ mice, *Stat3* ^*fl/+*^ *;Osx* ^*Cre*^ mice and *Stat3* ^*fl/fl*^ mice.
**Figure S4:** (A) H&E staining of mandibular first molars from 1‐week‐old and 3‐week‐old *Stat3* ^*fl/fl*^ *;Osx* ^*Cre*^ and *Stat3* ^*fl/fl*^ mice. (B) Quantitative analysis of pre‐dentine width and dentine width in 1‐ and 3‐week‐old mice from H&E staining images, *n* = 3. Error bars represent mean ± SD. *p < 0.05; **p < 0.01.
**Table S1:** CellRank‐identified driver genes for pre‐odontoblasts.
**Table S2:** SCENIC regulon specificity scores of pre‐odontoblasts.

## Data Availability

All the study data are included in the article or Supporting Information. Other data and genetic materials used in this article are available upon request to the corresponding authors.
