# Supplementary material for: Single‐Cell Virtual Perturbation Screening Identifies STAT3 as a Key Regulator of Dentinogenesis
Source: Cell Prolif. 2026 Apr 15:e70203. Online ahead of print. doi: 10.1111/cpr.70203 (PMC13325875; doi:10.1111/cpr.70203)
Supplement: Supplementary file 1 — Data S1: Supplementary experimental procedures. Figure S1: Supplementary analyses. (A) Leiden clusters. (B) Supplementary spatial distribution overview. (C) CytoTRACE2 scores across clusters. (D) Additional marker gene expression patterns. (E) Marker gene expression patterns of mesenchymal subclusters. (F) CytoTRACE2 scores across clusters of mesenchymal subclusters. (G) CellRank circular projection. (H) Dynamic STAT3 expression along pseudotime. Figure S2: (A) Micro‐CT images with colour mapping based on enamel density in mandibular molars from 4‐week‐old Stat3 fl/fl ;Osx Cre and Stat3 fl/fl mice. (B) Quantitative analysis of enamel thickness in mandibular first molars from 4‐week‐old mice, n = 5. Error bars represent mean ± SD. Ns, not significant (p > 0.05). Figure S3: Micro‐CT images of mandibular molars from 3‐, 4‐ and 8‐week‐old Stat3 fl/fl ;Osx Cre mice, Stat3 fl/+ ;Osx Cre mice and Stat3 fl/fl mice. Figure S4: (A) H&E staining of mandibular first molars from 1‐week‐old and 3‐week‐old Stat3 fl/fl ;Osx Cre and Stat3 fl/fl mice. (B) Quantitative analysis of pre‐dentine width and dentine width in 1‐ and 3‐week‐old mice from H&E staining images, n = 3. Error bars represent mean ± SD. *p < 0.05; **p < 0.01. Table S1: CellRank‐identified driver genes for pre‐odontoblasts. Table S2: SCENIC regulon specificity scores of pre‐odontoblasts. [file CPR-9999-e70203-s001.docx]

**Single-Cell Virtual Perturbation Screening Identifies STAT3 as a Key Regulator of Dentinogenesis**

Yanfei Zhu^1^, Hongyuan Xu^1^, Zijian Zhang^1^, Siyuan Sun^1^, Zihan Huang^1^, Xin Gao^1^, Houwen Pan^1^, Xiangru Huang^1^, Yuanqi Liu^1^, Xinyu Wang^1^, Hanbin Jia^1^, Qinggang Dai^2*^, Lingyong Jiang^1*^

**Supplementary experimental procedures**

***Quantitative reverse transcriptase polymerase chain reaction (qRT-PCR)***

Total RNA was isolated from hDPCs using TRIzol reagent (Sigma-Aldrich, MO, USA) and was converted into complementary DNA (cDNA) through reverse transcription utilizing the PrimeScript RT Master Mix (Takara Bio Inc., Shiga, Japan). qRT-PCR was conducted using the Bio-Rad CFX96 Real-Time PCR Detection System. The primer sequences were listed as follows:

Human-*STAT3* F: CAGCAGCTTGACACACGGTA

Human-*STAT3* R: AAACACCAAAGTGGCATGTGA

Human-*ALP* F: ACTGGTACTCAGACAACGAGAT

Human-*ALP* R: ACGTCAATGTCCCTGATGTTATG

Human-*DSPP* F: GCATTTGGGCAGTAGCATGG

Human-*DSPP* R: CTGACACATTTGATCTTGCTAGGAG

Human-*DMP1* F: CTCCGAGTTGGACGATGAGG

Human-*DMP1* R: TCATGCCTGCACTGTTCATTC

Human-*BGLAP* F: CACTCCTCGCCCTATTGGC

Human-*BGLAP* R: CCCTCCTGCTTGGACACAAAG

Human-*WNT2B* F: GGGGCACGAGTGATCTGTG

Human-*WNT2B* R: GCATGATGTCTGGGTAACGCT

Human-*CTNNB1* F: AAAGCGGCTGTTAGTCACTGG

Human-*CTNNB1* R: CGAGTCATTGCATACTGTCCAT

Human-*AXIN2* F: CAACACCAGGCGGAACGAA

Human-*AXIN2* R: GCCCAATAAGGAGTGTAAGGACT

Human-*LEF1* F: AGAACACCCCGATGACGGA

Human-*LEF1* R: GGCATCATTATGTACCCGGAAT

Human-*SOST* F: ACACAGCCTTCCGTGTAGTG

Human-*SOST* R: GGTTCATGGTCTTGTTGTTCTCC

Human-*GAPDH* F: TCATTGACCTCAACTACATG

Human-*GAPDH* R: TCGCTCCTGGAAGATGGTGAT

***Western blotting***

Total proteins were extracted from hDPCs using SDS-PAGE lysis buffer (Takara Bio Inc., Shiga, Japan) supplemented with protease inhibitor (Bimake, TX, USA) and phosphatase inhibitor (Bimake, TX, USA). Cell lysates were centrifuged at 12,000×g for 10 min at 4°C, and the protein-containing supernatant was collected. Subsequently, 20 μg of protein samples were separated on 10% SDS-PAGE gels and transferred onto PVDF membranes for western blotting analysis following standard procedures. The following primary antibodies were used: rabbit monoclonal anti-STAT3 (Abcam, Cambridge, UK, Cat.ab68153, 1:1000), rabbit monoclonal anti-p-STAT3 (CST, MA, USA, Cat.9145, 1:1000), rabbit monoclonal anti-WNT2B (ABclonal, Wuhan, China, Cat.A19554, 1:1000), and rabbit monoclonal anti-GAPDH (CST, MA, USA, Cat.2118, 1:1000).

***Hematoxylin and eosin (H&E), immunohistochemistry, and immunofluorescence staining***

The mandibles were fixed in 4% paraformaldehyde (PFA) for 24 h and subsequently decalcified in 0.5 M ethylenediaminetetraacetic acid (EDTA, pH 7.6) at 4°C for 4 weeks. Then the specimens were dehydrated through a graded ethanol series, embedded in paraffin, and sectioned at 5 μm thickness. For H&E staining, the slides were processed according to the manufacturer's protocol (Solarbio Science & Technology Inc., Beijing, China). For immunohistochemical staining, slides were deparaffinized, rehydrated through a graded alcohol series, and treated with 3% hydrogen peroxide (H_2_O_2_) to quench endogenous peroxidase activity. Afterward, antigen retrieval was performed by heating the sections in sodium citrate buffer at 99°C for 20 min, followed by blocking with 5% bovine serum albumin (BSA) for 30 min at room temperature. The sections were then incubated with rabbit monoclonal anti-STAT3 primary antibody (Abcam, Cambridge, UK, Cat.ab68153, 1:1000) at 4 °C overnight. After washing, sections were incubated with an anti-rabbit secondary antibody for 1 h at room temperature, and the immunoreactivity was visualized using an ABC kit (Vector Laboratories, CA, USA). For immunofluorescence staining, sections were incubated with the following primary antibodies: rabbit monoclonal anti-STAT3 (Abcam, Cambridge, UK, Cat.ab68153, 1:200), rabbit monoclonal anti-SP7 (Abcam, Cambridge, UK, Cat.ab209484, 1:200), and rabbit polyclonal anti-DSPP (Absin, Shanghai, China, Cat.abs154960, 1:200). The secondary antibodies included: goat anti-rabbit IgG Alexa Fluor 555 (Abcam, Cambridge, UK, Cat.ab150078, 1:1000) and goat anti-rabbit IgG Alexa Fluor 488 (Abcam, Cambridge, UK, Cat.ab150077, 1:1000). Nuclei were counterstained with DAPI. Fluorescent images were acquired using an inverted fluorescence microscope (IX83, Olympus, Tokyo, Japan).

***Micro-computed tomography (Micro-CT) analysis***

The harvested mandibles from mice were fixed in 4% PFA for 24 h at 4℃ and subsequently stored in 1×PBS prior to Micro-CT scanning. The mandibles were scanned by the SkyScan1176 (Bruker, Kontich, Belgium) with a voxel size of 5 μm and medium resolution for quantitative analysis.

***Calcein-alizarin red S (ARS) double labeling***

Mice were intraperitoneally injected with 20 mg/kg calcein (Sigma-Aldrich, MO, USA, Cat.C0875-5G; 1 mg/ml in 2% NaHCO3 solution) on day 0 and 40 mg/kg ARS (Sigma-Aldrich, MO, USA, Cat.A5533-25G; 2 mg/ml in distilled H_2_O) on day 5. The mice were euthanized 48 h after the second injection. The mandibles were fixed in 4% PFA, dehydrated through a graded ethanol series, and embedded in polymethylmethacrylate (PMMA). The specimens were cut into 10-μm-thick sections using a hard tissue microtome (Leica Microsystems, Wetzlar, Germany). The fluorescence-labeled images were captured using LSCM (Leica Microsystems, Wetzlar, Germany). Dentin formation rate was quantified by measuring mineral apposition rate (MAR).

***Gene knockdown and overexpression***

For *STAT3* knockdown, a shRNA targeting *STAT3* and a control shRNA were synthesized by Hanheng Biotechnology Inc. (Shanghai, China). HDPCs were seeded in 6-well plates at a density of 3×10^5^ cells per well in α-MEM for 18 h, then hDPCs were transduced with the lentiviruses in the presence of 6-8 μg/mL Polybrene (Hanheng Biotechnology Inc., Shanghai, China) for 24 h, followed by selection with 2 μg/mL puromycin (Hanheng Biotechnology Inc., Shanghai, China). Knockdown efficiency was confirmed through qRT-PCR and Western blotting assays.

For *WNT2B* overexpression, lentiviral vectors encoding *WNT2B*-GFP or GFP control were constructed by Hanheng Biotechnology Inc. (Shanghai, China). hDPCs were transduced with the lentiviruses for 24 h following the manufacturer’s protocol (Hanheng Biotechnology Inc., Shanghai, China). Overexpression efficiency was validated through qRT-PCR and Western blotting assays. The experimental design included the following four groups:

(1) DMSO + Lv-*CTRL* (vehicle control with empty vector lentivirus);

(2) DMSO + Lv-*WNT2B* (vehicle control with *WNT2B*-overexpressing lentivirus);

(3) AG490 + Lv-*CTRL* (STAT3 inactivation with empty vector lentivirus);

(4) AG490 + Lv-*WNT2B* (STAT3 inactivation with *WNT2B*-overexpressing lentivirus).

***Cell Counting Kit-8 (CCK-8) assay***

To evaluate cell proliferation capacity using CCK-8 assay, hDPCs were seeded in 96-well plates at a density of 4×10^3^ cells per well and cultured in α-MEM medium. The cells were then exposed to 10% CCK-8 reagent (Beyotime Biotechnology Inc., Shanghai, China) diluted in the culture medium, and incubated for 1 h at 37°C. The optical density (OD) value was quantified at 450 nm using a microplate spectrophotometer.

***5-Ethynyl-2’-deoxyuridine (EdU) cell proliferation assay***

EdU assay was performed using the EdU Cell Proliferation Kit (Beyotime Biotechnology Inc., Shanghai, China) according to the manufacturer's protocol. hDPCs grown on coverslips were incubated with α-MEM containing 10μM EdU for 6 h at 37°C, followed by fixation with 4% PFA for 20 min. EdU-positive cells were visualized by reacting with Apollo® fluorescent dye for 30 min. Cell nuclei were counterstained with Hoechst 33342 for 10 min at room temperature. Images were captured using an inverted fluorescence microscope (IX83, Olympus, Tokyo, Japan).

***Alkaline phosphatase (ALP) and ARS staining***

For odontoblast differentiation induction, hDPCs were seeded in 12-well plates at a density of 2×10^5^ cells per well and cultured in odontogenic induction medium (Cyagen Biosciences Inc., CA, USA). ALP activity was determined using a staining kit (Beyotime Biotechnology Inc., Shanghai, China) on day 7 of induction. Mineralization was evaluated by ARS staining (Beyotime Biotechnology Inc., Shanghai, China) on day 21 of induction.

***RNA-Seq and transcriptomic analysis***

Total RNA was isolated from hDPCs transduced with either *STAT3-*knockdown lentivirus or control EGFP-expressing lentivirus using TRIzol reagent. cDNA libraries were constructed using the Illumina TruSeq RNA sample preparation kit (Illumina, San Diego, CA, USA). Raw sequencing reads were subjected to quality control using FastQC to ensure high-quality data. The cleaned reads were then aligned to the human reference genome using TopHat version 1.4.1 with default parameters optimized for splice junction discovery. Gene expression levels were quantified as fragments per kilobase of transcript per million mapped reads (FPKM) using Cufflinks version 1.3.0. Differential gene expression analysis between *STAT3*-knockdown hDPCs and the control groups was conducted using Cuffdiff, with a significance threshold set at false discovery rate (FDR) <0.05.

Functional annotation and enrichment analysis of the differentially expressed genes (DEGs) were performed using the DAVID online tool. Gene Ontology (GO) terms were assigned to the DEGs, and the top enriched GO categories were selected based on their statistical significance (*P* < 0.05). This analysis provided insights into the biological processes, molecular functions, and cellular components significantly associated with *STAT3* deletion in hDPCs. Pathway analysis was performed using the Kyoto Encyclopedia of Genes and Genomes (KEGG) database. The DEGs were mapped to KEGG pathways to identify significantly enriched signaling pathways and biological processes. Pathways with *P* value < 0.05 were considered statistically significant.

***Chromatin immunoprecipitation (ChIP) assay***

ChIP assays were performed in C3H10T1/2 cells using the EZ-Magna ChIP kit (Millipore, MA, USA) according to the manufacturer's protocol. C3H10T1/2 cells cultured in 10 cm dishes at 80-90% confluency were cross-linked with 1% formaldehyde for 10 min at room temperature, followed by glycine quenching. Chromatin was enzymatically digested using micrococcal nuclease to generate DNA fragments ranging from 250 to 500 bp. Immunoprecipitation was carried out using rabbit monoclonal anti-STAT3 antibody (CST, MA, USA, Cat.12640, 1:50) with IgG (Millipore, MA, USA) serving as the negative control. Precipitated DNA was analyzed by qRT-PCR.

***Luciferase reporter assay***

The cDNA of *STAT3* was cloned into a phage-based plasmid. The *WNT2B* promoter was synthesized and subsequently inserted into PGL3-based luciferase reporter, generating the *WNT2B*-Luc reporter construct. A mutant *WNT2B* promoter with deletion of the predicted STAT3 binding site (in the promoter -2723 to -2713 bp region) was synthesized and subcloned into the PGL3 backbone to create the *WNT2B*-mu-Luc reporter. The constitutively active form of STAT3 (STAT3-C) and dominant-negative mutant of STAT3 (STAT3-DN) were kindly provided by Dr. Feng from Shanghai Institutes for Biological Sciences, Chinese Academy of Sciences.

HEK293T cells were plated in 24-well plates at a density of 2×10^5^ cells per well and cultured for 18 h to reach optimal confluency. Transfection was performed using Lipofectamine 8000 transfection reagent (Beyotime Biotechnology Inc., Shanghai, China) according to an established protocol. Each well received a luciferase reporter plasmid (either *WNT2B*-Luc or *WNT2B*-mut-Luc) along with renilla plasmid, and one of the following: STAT3, STAT3-C (constitutively active form), or STAT3-DN (dominant-negative form). To ensure consistent total DNA amounts across all wells, empty vector plasmids were added as needed. After 48 h of transfection, cells were lysed, and luciferase activity in the supernatant was measured using the Dual-Luciferase Reporter Assay System (Promega, WI, USA). Firefly luciferase activity was normalized to renilla luciferase activity for transfection efficiency control.

**Supplementary Figure**


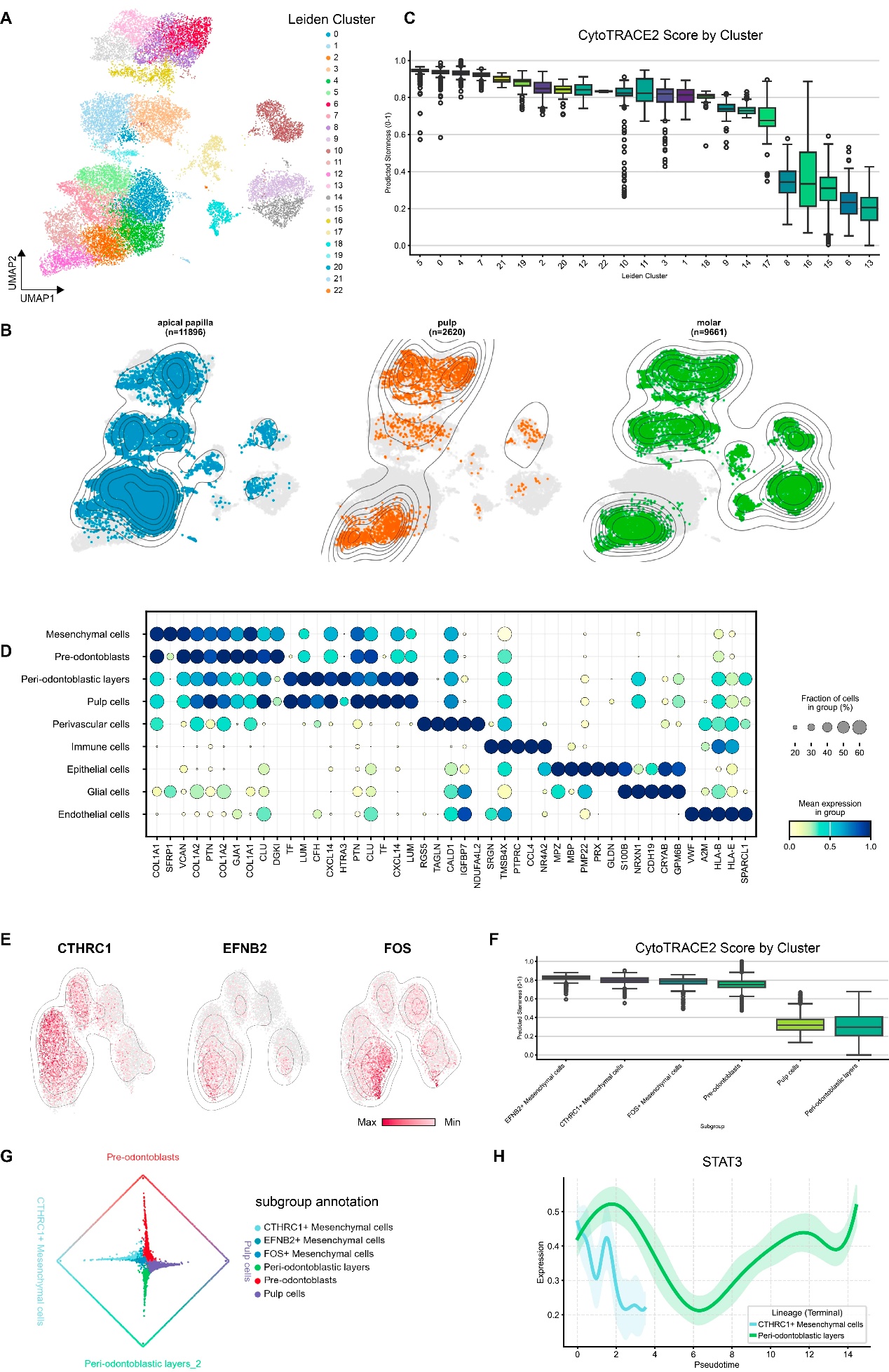


**Figure S1.** Supplementary analyses. **(A)** Leiden clusters. **(B)** Supplementary spatial distribution overview. **(C)** CytoTRACE2 scores across clusters. **(D)** Additional marker gene expression patterns. **(E)** Marker gene expression patterns of mesenchymal subclusters. **(F)** CytoTRACE2 scores across clusters of mesenchymal subclusters. **(G)** CellRank circular projection. **(H)** Dynamic STAT3 expression along pseudotime.


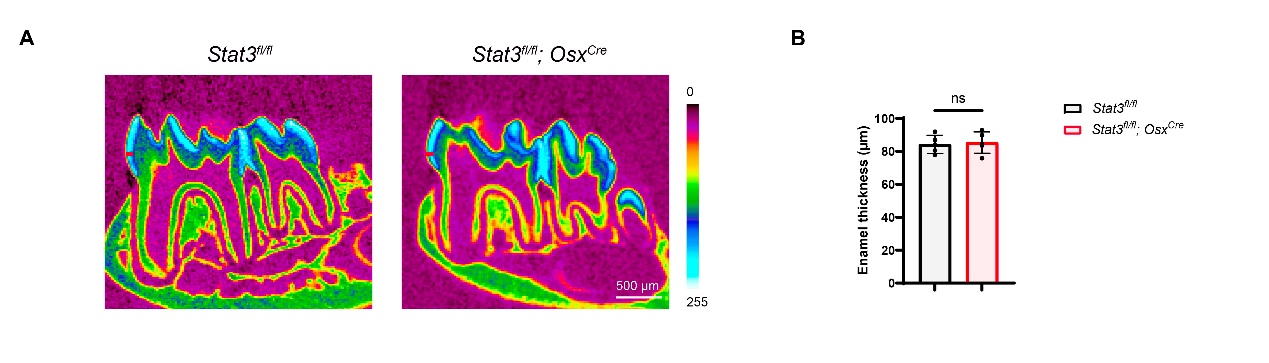


**Figure S2.** **(A)** Micro-CT images with color mapping based on enamel density in mandibular molars from 4-week-old *Stat3^fl/fl^;Osx^Cre^* and *Stat3^fl/fl^* mice. **(B)** Quantitative analysis of enamel thickness in mandibular first molars from 4-week-old mice, n = 5. Error bars represent mean ± SD. Ns, not significant (P > 0.05).


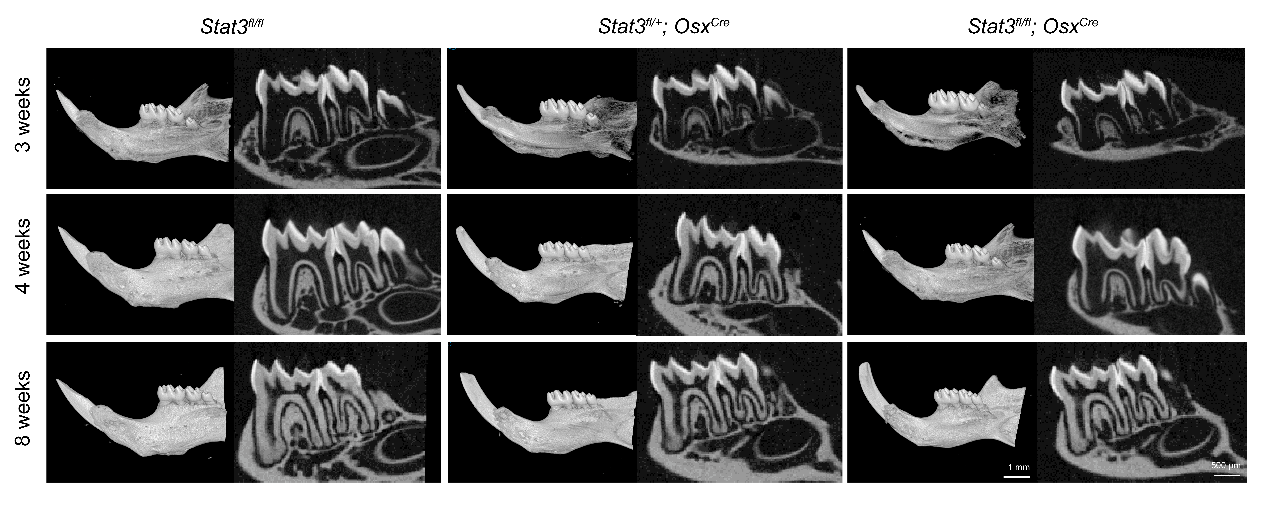


**Figure S3.** Micro-CT images of mandibular molars from 3-, 4-, and 8-week-old *Stat3^fl/fl^;Osx^Cre^* mice, *Stat3^fl/+^;Osx^Cre^* mice, and *Stat3^fl/fl^* mice.


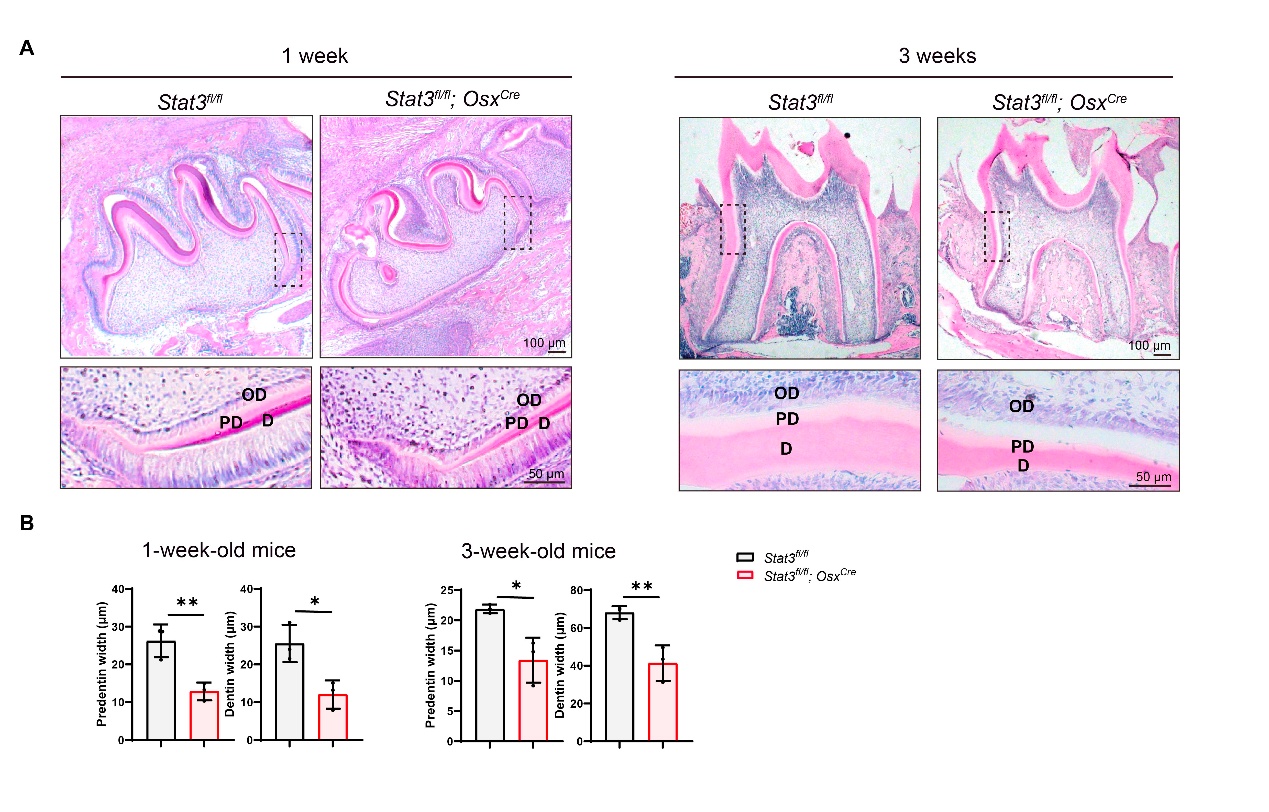


**Figure S4. (A)** H&E staining of mandibular first molars from 1-week-old and 3-week-old *Stat3^fl/fl^;Osx^Cre^* and *Stat3^fl/fl^* mice. **(B)** Quantitative analysis of pre-dentin width and dentin width in 1- and 3-week-old mice from H&E staining images, n = 3. Error bars represent mean ± SD. **P* < 0.05; ***P* < 0.01.

**Supplementary Table**

**Table. S1** CellRank-identified driver genes for pre-odontoblasts.

|  | **corr** | **pval** | **qval** | **ci_low** | **ci_high** |
| --- | --- | --- | --- | --- | --- |
| **DGKI** | 0.215051494 | 1.96E-109 | 1.47E-106 | 0.196602918 | 0.233347853 |
| **FBN2** | 0.196022785 | 9.06E-91 | 2.47E-88 | 0.177429746 | 0.214475938 |
| **COL1A1** | 0.19479413 | 1.25E-89 | 3.12E-87 | 0.176192248 | 0.213256933 |
| **SLITRK5** | 0.179419163 | 4.94E-76 | 8.23E-74 | 0.160711595 | 0.197997864 |
| **KCNQ1OT1** | 0.173897916 | 1.91E-71 | 2.73E-69 | 0.15515464 | 0.192516038 |
| **COL25A1** | 0.145449814 | 3.15E-50 | 2.42E-48 | 0.126541346 | 0.164252622 |
| **PIEZO2** | 0.123635479 | 1.23E-36 | 4.97E-35 | 0.104621634 | 0.142558974 |
| **TPM1** | 0.114158168 | 1.93E-31 | 5.97E-30 | 0.095104294 | 0.133128427 |
| **NCKAP5** | 0.113187483 | 6.22E-31 | 1.85E-29 | 0.094129705 | 0.132162337 |
| **DST** | 0.11000027 | 2.70E-29 | 7.05E-28 | 0.090929933 | 0.128989961 |
| **CACNB4** | 0.104607981 | 1.24E-26 | 2.72E-25 | 0.085517292 | 0.123621886 |
| **NTRK3** | 0.102845929 | 8.62E-26 | 1.82E-24 | 0.083748834 | 0.121867506 |
| **STAT3** | 0.102140302 | 1.86E-25 | 3.81E-24 | 0.083040675 | 0.121164918 |
| **BCAT1** | 0.098825723 | 6.31E-24 | 1.21E-22 | 0.079714462 | 0.117864358 |
| **LINC00662** | 0.095371066 | 2.20E-22 | 3.70E-21 | 0.076248133 | 0.114423866 |
| **ARHGAP28** | 0.094284018 | 6.55E-22 | 1.06E-20 | 0.075157506 | 0.11334118 |
| **VLDLR** | 0.09388906 | 9.70E-22 | 1.55E-20 | 0.07476126 | 0.112947796 |
| **ATP8A1** | 0.090498549 | 2.65E-20 | 3.98E-19 | 0.071359937 | 0.109570549 |
| **HBB** | 0.087713045 | 3.67E-19 | 5.05E-18 | 0.068565884 | 0.106795614 |
| **ADAMTS9** | 0.086408742 | 1.22E-18 | 1.58E-17 | 0.067257681 | 0.105496157 |
| **SLC2A13** | 0.085730052 | 2.27E-18 | 2.88E-17 | 0.066576987 | 0.104819962 |
| **SBSPON** | 0.085465359 | 2.88E-18 | 3.61E-17 | 0.066311517 | 0.104556238 |
| **HAPLN1** | 0.084814047 | 5.18E-18 | 6.39E-17 | 0.065658306 | 0.103907298 |
| **CXXC4** | 0.083975447 | 1.10E-17 | 1.31E-16 | 0.064817285 | 0.103071728 |
| **MT-ATP6** | 0.083960946 | 1.11E-17 | 1.33E-16 | 0.064802742 | 0.103057279 |
| **PTCH1** | 0.083430695 | 1.78E-17 | 2.07E-16 | 0.064270974 | 0.102528929 |
| **MMS22L** | 0.082590923 | 3.71E-17 | 4.22E-16 | 0.063428823 | 0.101692148 |
| **PCOLCE2** | 0.081618437 | 8.65E-17 | 9.23E-16 | 0.062453616 | 0.100723091 |
| **VLDLR-AS1** | 0.080663893 | 1.96E-16 | 2.03E-15 | 0.061496436 | 0.099771876 |
| **MYO3A** | 0.079639294 | 4.69E-16 | 4.70E-15 | 0.060469047 | 0.098750813 |
| **MEG8** | 0.07829625 | 1.44E-15 | 1.40E-14 | 0.059122408 | 0.097412343 |
| **SELP** | 0.077127535 | 3.78E-15 | 3.55E-14 | 0.057950621 | 0.096247551 |
| **GREB1L** | 0.076724964 | 5.24E-15 | 4.87E-14 | 0.057547005 | 0.09584632 |
| **WAS** | 0.076239885 | 7.77E-15 | 7.12E-14 | 0.057060673 | 0.095362846 |
| **TNN** | 0.075851075 | 1.06E-14 | 9.63E-14 | 0.056670866 | 0.094975316 |
| **MT-CYB** | 0.075807675 | 1.10E-14 | 9.94E-14 | 0.056627355 | 0.094932059 |
| **TRDN** | 0.075533347 | 1.37E-14 | 1.23E-13 | 0.056352328 | 0.094658631 |
| **CADPS** | 0.073948918 | 4.81E-14 | 4.05E-13 | 0.054763916 | 0.09307934 |
| **LGI2** | 0.071857736 | 2.42E-13 | 1.92E-12 | 0.052667626 | 0.090994793 |
| **HRASLS** | 0.071443643 | 3.32E-13 | 2.61E-12 | 0.052252542 | 0.090581994 |
| **ELL2** | 0.06939382 | 1.54E-12 | 1.14E-11 | 0.05019791 | 0.08853848 |
| **ADPRHL1** | 0.069296446 | 1.65E-12 | 1.21E-11 | 0.050100311 | 0.088441401 |
| **ADAMTS9-AS1** | 0.06833056 | 3.34E-12 | 2.38E-11 | 0.049132218 | 0.087478428 |
| **CCDC85A** | 0.067478163 | 6.18E-12 | 4.27E-11 | 0.048277904 | 0.086628572 |
| **CADM1** | 0.06515104 | 3.19E-11 | 2.11E-10 | 0.04594569 | 0.084308245 |
| **RAMP1** | 0.064673292 | 4.44E-11 | 2.87E-10 | 0.045466923 | 0.083831866 |
| **AMBN** | 0.062871422 | 1.51E-10 | 9.24E-10 | 0.043661288 | 0.082035082 |
| **SORL1** | 0.06207083 | 2.57E-10 | 1.53E-09 | 0.042859064 | 0.08123671 |
| **SLC38A1** | 0.06197368 | 2.74E-10 | 1.62E-09 | 0.042761717 | 0.081139827 |
| **MIA** | 0.061308393 | 4.24E-10 | 2.44E-09 | 0.042095095 | 0.080476363 |
| **LRRTM3** | 0.060598139 | 6.72E-10 | 3.75E-09 | 0.041383434 | 0.079768037 |
| **AFF2** | 0.060256805 | 8.37E-10 | 4.60E-09 | 0.041041431 | 0.079427623 |
| **ZNF793** | 0.059873881 | 1.07E-09 | 5.79E-09 | 0.040657762 | 0.079045725 |
| **SHANK2** | 0.059116193 | 1.73E-09 | 9.17E-09 | 0.039898616 | 0.078290051 |
| **NAV2** | 0.059005158 | 1.86E-09 | 9.80E-09 | 0.039787369 | 0.078179309 |
| **ENPP2** | 0.056361468 | 9.47E-09 | 4.69E-08 | 0.037138778 | 0.075542464 |

**Table. S2** SCENIC regulon specificity scores of pre-odontoblasts.

|  | **FOS+ Mesenchymal cells** | **Peri-odontoblastic**  **layers** | **EFNB2+ Mesenchymal**  **cells** | **CTHRC1+ Mesenchymal**  **cells** | **Pre-odontoblasts** | **Pulp cells** |
| --- | --- | --- | --- | --- | --- | --- |
| **ELF1(+)** | 0.30127911 | 0.27878373 | 0.30959541 | 0.34561767 | 0.36236692 | 0.26826006 |
| **MBD2(+)** | 0.28942651 | 0.28508728 | 0.32634767 | 0.33248046 | 0.34512294 | 0.28673791 |
| **ETS2(+)** | 0.28305984 | 0.30974008 | 0.30592293 | 0.33757162 | 0.34288758 | 0.29438868 |
| **MXI1(+)** | 0.28774673 | 0.30840292 | 0.31348472 | 0.32971995 | 0.33749936 | 0.28774919 |
| **STAT3(+)** | 0.31441725 | 0.29191666 | 0.32849589 | 0.30971494 | 0.33410093 | 0.27642877 |
| **KLF4(+)** | 0.30112481 | 0.30721943 | 0.33214174 | 0.32590707 | 0.33279253 | 0.27799148 |
| **CEBPG(+)** | 0.28276272 | 0.33414046 | 0.30139857 | 0.33442948 | 0.32611371 | 0.32086892 |
| **IRF1(+)** | 0.32590651 | 0.27981544 | 0.33275239 | 0.32667263 | 0.32575247 | 0.26563694 |
| **STAT1(+)** | 0.28916266 | 0.3287005 | 0.32450553 | 0.33858275 | 0.32557444 | 0.29070975 |
| **MITF(+)** | 0.26979138 | 0.3310517 | 0.28328181 | 0.29737904 | 0.32500022 | 0.35170793 |
| **EGR1(+)** | 0.30691086 | 0.29786608 | 0.34493815 | 0.3382087 | 0.32489466 | 0.25644774 |
| **NR3C1(+)** | 0.31060207 | 0.33404626 | 0.33967942 | 0.3225013 | 0.32180311 | 0.29251383 |
| **ETV4(+)** | 0.28942814 | 0.26262413 | 0.30931453 | 0.35439628 | 0.31982757 | 0.24038815 |
| **ZEB1(+)** | 0.30563534 | 0.3148024 | 0.33021836 | 0.33943081 | 0.31844168 | 0.29554064 |
| **E2F1(+)** | 0.2898667 | 0.27120034 | 0.35034219 | 0.36279452 | 0.31627016 | 0.24304333 |
| **PRDM1(+)** | 0.27082864 | 0.30332449 | 0.2902697 | 0.31814713 | 0.31540826 | 0.3215065 |
| **IRX3(+)** | 0.24468524 | 0.29213867 | 0.26375651 | 0.27094068 | 0.31486817 | 0.2975804 |
| **ARID3A(+)** | 0.28989509 | 0.30440331 | 0.3131206 | 0.31701604 | 0.31325401 | 0.28806241 |
| **ZNF891(+)** | 0.29584864 | 0.2639708 | 0.36705337 | 0.36152828 | 0.31241892 | 0.23066472 |
| **CEBPB(+)** | 0.29057282 | 0.36890938 | 0.30897849 | 0.31013675 | 0.31199995 | 0.34451474 |
| **IRF4(+)** | 0.2819052 | 0.2767729 | 0.31863911 | 0.3283131 | 0.31148121 | 0.2650895 |
| **CEBPD(+)** | 0.29834553 | 0.34514544 | 0.31652485 | 0.30806122 | 0.31120784 | 0.33828872 |
| **CHURC1(+)** | 0.30382575 | 0.30983015 | 0.34812709 | 0.31316672 | 0.31040249 | 0.30905043 |
| **ETS1(+)** | 0.28858663 | 0.35158011 | 0.3181253 | 0.31071109 | 0.31025771 | 0.31601568 |
| **DBP(+)** | 0.2880162 | 0.35825885 | 0.30988212 | 0.30964549 | 0.31012639 | 0.33043754 |
| **ARID5B(+)** | 0.30141186 | 0.32065909 | 0.31396177 | 0.31985165 | 0.30993527 | 0.30748029 |
| **ATF3(+)** | 0.31094601 | 0.2932945 | 0.31444757 | 0.31288048 | 0.3092826 | 0.29773829 |
| **SP6(+)** | 0.3051962 | 0.28053215 | 0.33347114 | 0.35543958 | 0.30925474 | 0.26615943 |
| **JUND(+)** | 0.30157568 | 0.34232478 | 0.31297452 | 0.3103029 | 0.3091143 | 0.34048801 |
| **ZNF660(+)** | 0.23015283 | 0.29379668 | 0.24247416 | 0.26563887 | 0.30384291 | 0.24221075 |
| **JUNB(+)** | 0.33941996 | 0.30280018 | 0.31746807 | 0.30489996 | 0.30287753 | 0.28068056 |
| **ETV1(+)** | 0.26259365 | 0.34935716 | 0.30287291 | 0.3085153 | 0.29954117 | 0.29618977 |
| **SOX18(+)** | 0.2831146 | 0.26953029 | 0.32510893 | 0.30747557 | 0.29750045 | 0.26841599 |
| **SOX11(+)** | 0.2610821 | 0.21871689 | 0.29872608 | 0.28419364 | 0.29681963 | 0.21588519 |
| **PRRX2(+)** | 0.35428135 | 0.30158778 | 0.34866057 | 0.31910182 | 0.29672558 | 0.28970615 |
| **HIC1(+)** | 0.23378901 | 0.2162555 | 0.22623535 | 0.26856597 | 0.29600031 | 0.23226779 |
| **DDIT3(+)** | 0.27751607 | 0.35420816 | 0.28865721 | 0.32034455 | 0.29164827 | 0.2878003 |
| **ZNF143(+)** | 0.28981169 | 0.30675336 | 0.30531158 | 0.30275997 | 0.29065056 | 0.30008535 |
| **GLIS3(+)** | 0.20728413 | 0.17968827 | 0.20070994 | 0.25379703 | 0.29020021 | 0.17698766 |
| **IRF7(+)** | 0.2459416 | 0.23665489 | 0.26345796 | 0.30660206 | 0.28984464 | 0.3075487 |
| **JUN(+)** | 0.3941682 | 0.30909103 | 0.30669262 | 0.29116346 | 0.28925957 | 0.27890655 |
| **HEY2(+)** | 0.28023703 | 0.35416416 | 0.3170847 | 0.32232196 | 0.28895071 | 0.28703586 |
| **NR2F2(+)** | 0.28149955 | 0.38212612 | 0.31141378 | 0.29097343 | 0.28833183 | 0.32860189 |
| **ZNF26(+)** | 0.25318655 | 0.27648746 | 0.28213915 | 0.32449128 | 0.28775488 | 0.23310378 |
| **VDR(+)** | 0.23024969 | 0.29019776 | 0.26568716 | 0.25594593 | 0.28716855 | 0.32359688 |
| **IRF9(+)** | 0.27762671 | 0.26184109 | 0.30459822 | 0.29068791 | 0.2868904 | 0.27060996 |
| **CREM(+)** | 0.25043524 | 0.23101545 | 0.27958128 | 0.28415605 | 0.2858859 | 0.22451764 |
| **RARB(+)** | 0.27720519 | 0.34646524 | 0.3043057 | 0.28540493 | 0.28426423 | 0.28693718 |
| **ATF1(+)** | 0.29168815 | 0.29578217 | 0.32914004 | 0.34846151 | 0.28097307 | 0.26176071 |
| **HEY1(+)** | 0.27368124 | 0.3976911 | 0.28634851 | 0.28807223 | 0.27755272 | 0.35161211 |
| **MSX1(+)** | 0.2635001 | 0.27814973 | 0.28860506 | 0.27248287 | 0.27350838 | 0.31425437 |
| **RUNX1(+)** | 0.27276403 | 0.30360215 | 0.2926513 | 0.27217897 | 0.27074615 | 0.27881271 |
| **HES1(+)** | 0.21399765 | 0.27927715 | 0.22111892 | 0.2412795 | 0.26839926 | 0.2457343 |
| **MYC(+)** | 0.24555957 | 0.24116835 | 0.26985522 | 0.2649951 | 0.26303129 | 0.21632354 |
| **SALL1(+)** | 0.29669203 | 0.34882345 | 0.2850216 | 0.24624479 | 0.26098154 | 0.32577284 |
| **TCF4(+)** | 0.32070746 | 0.26744974 | 0.35506465 | 0.27373932 | 0.25920458 | 0.25364702 |
| **AR(+)** | 0.23973961 | 0.24460474 | 0.27279528 | 0.24637294 | 0.25709457 | 0.23780628 |
| **HEYL(+)** | 0.22246801 | 0.28699207 | 0.23361149 | 0.23498863 | 0.25446281 | 0.21992635 |
| **NR2F1(+)** | 0.25006973 | 0.37759192 | 0.28015868 | 0.24989754 | 0.25277231 | 0.41414354 |
| **FOSB(+)** | 0.41055488 | 0.32861182 | 0.30416032 | 0.26278878 | 0.25055982 | 0.26362657 |
| **MAFB(+)** | 0.23219983 | 0.24004984 | 0.26063442 | 0.23936751 | 0.24834471 | 0.23425321 |
| **ZNF154(+)** | 0.32864724 | 0.2153346 | 0.25876629 | 0.24016212 | 0.24756128 | 0.2121873 |
| **NFIB(+)** | 0.23623775 | 0.20720639 | 0.2695495 | 0.23628916 | 0.24610834 | 0.19770098 |
| **BCL6(+)** | 0.2088711 | 0.21146134 | 0.22113261 | 0.23278083 | 0.24144783 | 0.21257292 |
| **FOS(+)** | 0.40933302 | 0.26508469 | 0.26186589 | 0.24838678 | 0.23990336 | 0.25902562 |
| **RELB(+)** | 0.23296703 | 0.21408963 | 0.29254818 | 0.24055203 | 0.22918765 | 0.20883352 |
| **ATF5(+)** | 0.22292851 | 0.23780443 | 0.2579254 | 0.22349504 | 0.21075301 | 0.20525565 |
| **KLF15(+)** | 0.18416718 | 0.19740606 | 0.18897954 | 0.19284185 | 0.19277658 | 0.21189775 |
